# 10‐Gingerol Alleviates Arsenic Trioxide‐Induced Cardiotoxicity: Mechanisms Involving the PI3K/AKT Pathway Revealed by Network Pharmacology and Experimental Validation

**DOI:** 10.1002/fsn3.72180

**Published:** 2026-07-28

**Authors:** Bin Zheng, Hui Yin, Jiabao Chen, Xiaodong Xin, Lan Yan, Chunhua Li, Hefei Wang, Hua Zhou

**Affiliations:** ^1^ School of Pharmacy Hebei University of Chinese Medicine Shijiazhuang Hebei China; ^2^ Hebei Technology Innovation Center of TCM Formula Preparations Shijiazhuang Hebei China; ^3^ Hebei Higher Education Applied Technology Research Center of TCM Development and Industrialization Shijiazhuang Hebei China; ^4^ Department of Pharmacy The First Affiliated Hospital of Hebei North University Zhangjiakou Hebei China; ^5^ Hebei Key Laboratory of Systems Biology and Gene Regulation Zhangjiakou Hebei China; ^6^ Affiliated Hospital of Hebei University of Chinese Medicine Shijiazhuang Hebei China; ^7^ Endocrinology and Metabolism Center, Henan Key Laboratory of Rare Diseases The First Affiliated Hospital, and College of Clinical Medicine of Henan University of Science and Technology Luoyang Henan China; ^8^ Henan Academy of Innovations in Medical Science Zhengzhou Henan China

**Keywords:** 10‐Gingerol, arsenic trioxide, cardiotoxicity, heart injury, PI3K/AKT pathway

## Abstract

Arsenic trioxide (ATO) is an effective chemotherapeutic agent but causes severe cardiotoxicity, which limits its clinical application. 10‐Gingerol (10Gin) is a major bioactive component of ginger, yet its cardioprotection and the mechanism underlying its effects against ATO‐induced heart injury (HI) have not been fully elucidated. This study integrated network pharmacology (NP) analysis, in vivo mouse models, and in vitro cell experiments to investigate the cardioprotection of 10Gin against ATO‐induced HI and its underlying mechanisms. NP analysis identified 32 overlapping therapeutic targets of 10Gin, ATO, and HI, among which the phosphoinositide 3‐kinase/protein kinase B (PI3K/AKT) pathway was the most significantly enriched cardiovascular‐related pathway. Our in vivo experimental results demonstrated that 10Gin treatment significantly ameliorated ATO‐induced histopathological injury and cardiac dysfunction in mice. Echocardiographic evaluation showed that 10Gin improved ATO‐induced systolic dysfunction, as evidenced by increased ejection fraction and fractional shortening. Specifically, 10Gin reduced serum levels of myocardial injury markers, enhanced endogenous antioxidant enzyme activities, decreased reactive oxygen species production, downregulated pro‐inflammatory cytokine mRNA expression, and inhibited cardiomyocyte apoptosis. In vitro experiments involving H9c2 cells and AC16 human cardiomyocytes showed that 10Gin enhanced cell viability, attenuated apoptosis and oxidative stress, thereby exerting cardioprotective effects. Moreover, 10Gin significantly increased the p‐PI3K/PI3K and p‐AKT/AKT ratios both in vivo and in vitro. Notably, the in vitro cardioprotection of 10Gin could be partly reversed by the PI3K inhibitor LY294002. These findings indicate that 10Gin alleviates ATO‐induced HI through antioxidant, anti‐inflammatory, and anti‐apoptotic mechanisms, which are likely mediated by PI3K/AKT activation, thereby supporting further investigation of 10Gin as a potential adjunctive candidate.

## Graphical abstract

Network pharmacology combined with in vivo and in vitro experiments identified PI3K/AKT signaling as a candidate pathway associated with the protective effects of 10‐Gingerol (10Gin) against arsenic trioxide (ATO)‐induced cardiotoxicity. 10Gin improved myocardial histopathology and cardiac systolic function in ATO‐treated mice, while attenuating oxidative stress, inflammation, and apoptosis in both animal and cardiomyocyte models. These effects were accompanied by restoration of PI3K and AKT phosphorylation, whereas the PI3K inhibitor LY294002 partially weakened the protective actions of 10Gin. These findings suggest that 10Gin attenuates ATO‐induced heart injury through antioxidant, anti‐inflammatory, and anti‐apoptotic effects involving PI3K/AKT signaling.

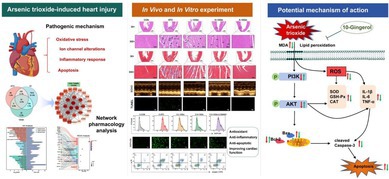

## 1 Introduction

Arsenic is a metalloid element that is widely distributed in water, soil, air, and rocks (Oremland and Stolz 2003). In the natural environment, it mainly exists in organic and inorganic forms (Oremland and Stolz 2003). Inorganic arsenic has been classified as a Group 1 human carcinogen by the International Agency for Research on Cancer, and its contamination in groundwater has become a global public health concern. It has been reported that in more than 70 countries (including China, India, and Argentina), arsenic concentrations in groundwater have exceeded the 10 μg/L safety threshold established by the World Health Organization, exposing hundreds of millions of people to the risk of chronic arsenic toxicity (Podgorski and Berg 2020). Chronic ingestion of excessive inorganic arsenic induces systemic toxic effects, resulting in serious injury to multiple organs and systems (Podgorski and Berg 2020). Numerous studies have shown that arsenic exposure, in addition to its well‐established cardiovascular toxicity, also induces hepatotoxicity (characterized by hepatic steatosis, inflammatory infiltration, and liver cirrhosis), nephrotoxicity (characterized by tubular epithelial injury, glomerular filtration dysfunction, and acute kidney injury), pulmonary toxicity (characterized by pulmonary fibrosis, airway remodeling, and impaired respiratory function), neurotoxicity, and reproductive toxicity (Li et al. 2025; Mochizuki 2019; Niu et al. 2025; Pan et al. 2024; Sanders et al. 2019; Xiao et al. 2024). Arsenic‐induced multiple organ injury is primarily driven by excessive reactive oxygen species (ROS) generation, activation of inflammatory cascades, induction of apoptosis, and redox homeostasis imbalance, which are also the core pathogenic mechanisms of arsenic in the injury across different tissues and organs (Ganie et al. 2023; Yang et al. 2024).

Despite the definite systemic toxicity of arsenic, the medicinal use of arsenic compounds has exceeded two thousand years (Paul et al. 2023). Arsenic trioxide (ATO) is a representative trivalent arsenic compound, which was historically used in the treatment of diseases including syphilis, tuberculosis, and psoriasis (Yan et al. 2024). In modern clinical practice, ATO is primarily used in the treatment of hematological malignancies such as acute promyelocytic leukemia (APL). Its anti‐tumor effects are related to multiple mechanisms, including selective induction of promyelocyte differentiation, promotion of tumor cell apoptosis, and inhibition of tumor angiogenesis (Davison et al. 2002; Liu et al. 2021). The National Comprehensive Cancer Network guidelines recommend the ATO plus all‐trans retinoic acid regimen as the first‐line treatment for newly diagnosed, relapsed, or refractory APL, with this combination yielding a 5‐year overall survival rate exceeding 90% in APL patients (Platzbecker et al. 2025; Pollyea et al. 2023). Encouragingly, ATO has also demonstrated favorable therapeutic efficacy in the studies of multiple solid malignancies, including lung cancer, liver cancer, and gastric cancer, thereby further broadening its potential clinical applications (Chen et al. 2023).

However, ATO is a highly toxic compound with a narrow therapeutic window, and the therapeutic concentration range significantly overlaps with its toxic range, which limits its widespread clinical application. Similar to inorganic arsenic exposure, ATO administration can also cause multiorgan toxicity, including cardiotoxicity, hepatotoxicity, and nephrotoxicity, among which cardiotoxicity is the most common and life‐threatening adverse reaction (Yan et al. 2024). The clinical manifestations of ATO toxicity‐induced heart injury (HI) are diverse, with mild cases presenting as asymptomatic electrocardiographic abnormalities (such as QT interval prolongation and arrhythmia), and severe cases progressing to heart failure, dilated cardiomyopathy, and even sudden cardiac death caused by torsades de pointes (Wei et al. 2024; Yan et al. 2024). Numerous studies have shown that the pathogenic mechanisms of HI caused by ATO toxicity involve oxidative stress, inflammatory cascade reactions, cell apoptosis, calcium homeostasis disorders, and ion channel remodeling (Li, Wan, et al. 2022; Sun et al. 2021; Vineetha and Raghu 2019; Yan et al. 2024). The cytoplasmic toxicity of ATO can directly damage mitochondria and the endoplasmic reticulum, leading to excessive ROS generation and redox imbalance, which in turn activates the NOD‐like receptor family pyrin domain‐containing 3 (NLRP3) inflammasome and nuclear factor‐kappa B (NF‐κB) pathway, triggering the massive release of pro‐inflammatory cytokines including tumor necrosis factor‐α (TNF‐α), interleukin‐6 (IL‐6), and IL‐1β (Dominic et al. 2022; Miller et al. 2002; Yan et al. 2024). The vicious cycle between oxidative stress and inflammation further impairs mitochondrial function, activates caspase‐dependent apoptotic cascades, and ultimately induces cardiomyocyte apoptosis, myocardial structural and electrical remodeling (Dominic et al. 2022; Elias‐Llumbet et al. 2024; Martins et al. 2022). Although the pathogenesis of ATO‐induced cardiotoxicity has been increasingly elucidated, there are still no approved myocardial protective agents for its prevention and treatment in clinical practice. The existing clinical intervention strategies are limited to symptomatic treatments (such as electrolyte supplementation and antiarrhythmic agents), which cannot fundamentally alleviate the HI caused by ATO (Yan et al. 2024). Thus, the screening of safe and effective drugs to alleviate HI caused by ATO remains an important clinical need.

Ginger ( *Zingiber officinale* Roscoe), an indispensable condiment in daily life, has also been used for millennia for its medicinal properties and contains numerous bioactive constituents (Garza‐Cadena et al. 2023). Among these constituents, gingerols are the primary bioactive compounds and the major contributors to its pungent flavor, which are characterized by a 3‐methoxy‐4‐hydroxyphenyl moiety. Based on the length of the aliphatic chain attached to this moiety, gingerols are classified as 6‐, 8‐, or 10‐Gingerol (Maghraby et al. 2023). 10‐Gingerol (10Gin, C_21_H_34_O_4_, MW: 350.49) has a longer carbon chain and exerts stronger biological activities, including anti‐inflammation, anti‐oxidation, anti‐apoptosis, myocardial protection, and inhibition of tumor cell growth (Garza‐Cadena et al. 2023). Our previous research has demonstrated that 10Gin dose‐dependently inhibits L‐type calcium channels, thereby attenuating myocardial calcium overload and ameliorating ischemic myocardial injury, which is consistent with the pathogenesis of ATO toxicity‐induced HI (Han et al. 2022). Furthermore, 10Gin can protect cardiomyocytes against hypoxia/reoxygenation injury through its antioxidant, anti‐inflammatory, and anti‐apoptotic effects (Zheng, Qi, et al. 2021). Based on the above findings, 10Gin may serve as a potential therapeutic agent for alleviating ATO toxicity‐induced HI. However, the specific cardioprotective effects of 10Gin against ATO toxicity‐induced HI have not yet been reported, and the underlying mechanisms remain unclear. Thus, the aim of this study is to verify the protection of 10Gin against ATO‐induced HI and elucidate the underlying mechanisms, thereby providing an experimental basis for its further investigation.

In summary, we hypothesized that 10Gin could alleviate ATO‐induced HI. To investigate this hypothesis, we integrated network pharmacology (NP) and experimental validation in vivo and in vitro to comprehensively evaluate the cardioprotection of 10Gin and elucidate its underlying mechanisms. NP is a powerful tool for predicting potential drug therapeutic targets based on systems biology and computational biology. NP links drugs to diseases by quantifying key regulatory nodes in biological networks (including key molecules, pathways, and functional modules) (Funari et al. 2026). Briefly, the potential therapeutic targets of 10Gin against HI caused by ATO and its main action signaling pathways were identified by NP analysis. The effects of 10Gin on myocardial injury biomarkers, histopathological changes, cardiac function, oxidative stress, inflammatory response, and apoptosis were systematically assessed using an in vivo mouse model and in vitro cell models. To further explore its underlying mechanisms, Western Blotting (WB) was used to detect the expression levels of proteins associated with the signaling pathways predicted by NP. This study provides experimental evidence supporting the further investigation of 10Gin as a potential adjunctive candidate for alleviating ATO‐induced cardiotoxicity.

## 2 Materials and Methods

### 2.1 Reagents and Materials

10Gin (Purity ≥ 98%, Catalog: BP0013) was obtained from Chengdu Biopurify Technology Development Co. Ltd. (Chengdu, China). ATO (Catalog: H20080665) for injection was purchased from Beijing Shuanglu Pharmaceutical Co. Ltd. (Beijing, China). LY294002 (Purity ≥ 99.95%, Catalog: HY‐10108) was purchased from MedChemExpress (MCE, NJ, USA). Unless otherwise stated, all scientific research reagents used in this study were purchased from Sigma‐Aldrich Co. Ltd., and the experimental test kits were purchased from Nanjing Jiancheng Bioengineering Institute (Nanjing, China).

### 2.2 NP Analysis

#### 2.2.1 Data Collection for Target Screening

Targets of 10Gin were obtained from the Traditional Chinese Medicine Systems Pharmacology (TCMSP) database. However, due to the lag in TCMSP updates, the Swiss Target Prediction (STP) and SuperPRED Target Prediction (SPTP) databases were employed to refine this target list. First, the canonical SMILES information of 10Gin was retrieved from the PubChem database. This retrieved SMILES information was then imported into the STP and SPTP databases for target prediction, where potential drug targets with a probability greater than 0.10 were selected. Duplicates were removed, and the remaining targets were merged to form the final set of potential targets for 10Gin. Targets for ATO were identified by searching the Comparative Toxicogenomics Database (CTD) using “arsenic trioxide” as the keyword, and results under the “Gene” category were exported. Targets associated with HI were retrieved from the GeneCards, OMIM, and DrugBank databases using the keyword “heart injury,” followed by merging of the targets from these databases and removal of duplicate entries. All target nomenclature was standardized through the UniProt database. Finally, the intersecting targets among 10Gin, ATO, and HI were identified as potential therapeutic targets for 10Gin and visualized using a Venn diagram. Furthermore, the intersecting targets were imported into Cytoscape 3.9.1 for further visualization. All the uniform resource locators of the databases used for target screening were summarized in Table S1 (Supporting Information 1, page 1).

#### 2.2.2 Construction of the Protein–Protein Interaction (PPI) Network

The intersecting targets of 10Gin, ATO, and HI, collected as described in Section 2.2.1, were imported into the STRING database (v12.0). The organism was set to *Homo sapiens* , and a minimum combined confidence score threshold > 0.4 was applied (Zhao et al. 2024). The layout of the PPI network was adjusted in the visualization interface to generate the final network diagram.

#### 2.2.3 Screening of Core Candidate Therapeutic Targets for 10Gin

The PPI network data obtained from the STRING database were imported into Cytoscape 3.9.1, and degree centrality (DC), closeness centrality (CCT), and betweenness centrality (BC) were calculated using the CytoNCA plugin (Qi et al. 2024). In the network, nodes with values of DC, CCT, and BC simultaneously exceeding the respective medians were identified as potential core therapeutic targets of 10Gin. Finally, these core targets were visualized as circular nodes and ordered in descending sequence based on their DC values.

#### 2.2.4 Kyoto Encyclopedia of Genes and Genomes (KEGG) and Gene Ontology (GO) Enrichment Analyses

The obtained 10Gin intersecting targets were imported into the Database for Annotation, Visualization and Integrated Discovery (DAVID) database. For the GO functional enrichment analysis, three types of GO annotations, namely biological process (BP), molecular function (MF), and cellular component (CC), were selected. Meanwhile, KEGG pathway enrichment analysis was conducted to identify significantly enriched biological pathways. GO functions and KEGG pathway terms with *p* < 0.01 were considered significantly enriched.

### 2.3 Animal Experiment Validation

#### 2.3.1 Animals and Treatment

Fifty 6‐week‐old male Kunming mice (22 ± 2 g) were obtained from the Experimental Animal Center of Hebei Medical University (Animal Production License No: SCXK (Ji) 2025‐005). Mice were acclimatized to a temperature (23°C ± 2°C) and humidity (55% ± 5%) controlled specific pathogen‐free environment under a 12 h light/dark cycle. Mice received a standard diet and water ad libitum, with routine replenishment of water and bedding every 2 days. The study protocols were approved by the Laboratory Animal Welfare and Ethics Committee of Hebei University of Chinese Medicine (Approval No: DWLL202506003). All procedures were performed in full compliance with both the institutional guidelines and the National Institute of Health Guide for the Care and Use of Laboratory Animals (NIH Publication No. 85‐23, revised 1996). After one week of acclimatization, all mice were randomly assigned to five groups (*n* = 10):

1. Solvent control group (CON)
2. ATO‐treated group (ATO)
3. Low‐dose 10Gin + ATO co‐treated group (L‐10Gin)
4. High‐dose 10Gin + ATO co‐treated group (H‐10Gin)
5. High‐dose 10Gin alone‐treated group (A‐10Gin)

Specifically, except for the CON group and A‐10Gin group, the HI model was established by intraperitoneal injection (IP) of ATO at a dose of 7.5 mg/kg per day for 2 weeks (Sun et al. 2021). For the L‐10Gin and H‐10Gin groups, 10Gin was administered via oral gavage at doses of 20 mg/kg and 40 mg/kg per day, respectively, to alleviate ATO‐induced HI (Han et al. 2022). The CON group received IP of an equal volume of 0.9% normal saline (NS) and oral gavage of 1% sodium carboxymethyl cellulose aqueous (CMC‐Na) in the same volume as that used for the 10Gin‐treated groups. The A‐10Gin group was given IP of 0.9% NS per day plus oral gavage of 10Gin at 40 mg/kg per day (Han et al. 2022). The A‐10Gin group was designed as the drug safety control group in the animal experiments of this study. Its core purpose was to validate the in vivo biosafety of 10Gin at the maximum administered dose in normal mice through parallel comparison with the CON group, thereby excluding the risk of inherent cardiac or systemic toxicity of the drug itself. The design of this study group is consistent with previous studies, using high‐dose therapeutic drugs as the dosage for the drug safety control group (Liu et al. 2020; Sun et al. 2021; Zheng, Yang, et al. 2021). Water intake, food intake, and changes in body weight were recorded daily throughout the experimental period for subsequent analysis (Table 1; this assay included 10 mice per group).

**TABLE 1:** Summary of general observations in mice.

General observations	CON	ATO	L‐10Gin	H‐10Gin	A‐10Gin
Initial body weight (g)	23.4 ± 1.8	23.2 ± 1.6	23.1 ± 2.4	23.3 ± 1.8	22.7 ± 1.9
Final body weight (g)	47.7 ± 4.3	33.4 ± 5.7^##^	38.3 ± 4.5	43.9 ± 5.3**	46.2 ± 3.2
Mean food consumption (g/mouse/day)	8.8 ± 1.5	6.1 ± 2.1^##^	6.8 ± 2.2	7.5 ± 2.4*	8.5 ± 1.9
Mean water consumption (mL/mouse/day)	22.3 ± 3.2	16.3 ± 3.9^##^	18.4 ± 5.3	21.2 ± 4.5*	22.9 ± 3.8
Mortality (ratio %)	0.0	0.0	0.0	0.0	0.0

*Note:* Values are given as mean ± SD (*n* = 10). Denote significance with ^##^ *p* < 0.01 vs. CON; *p < 0.05, **p < 0.01 vs. ATO.

10Gin was prepared using a 1% CMC‐Na solution (Han et al. 2022). ATO was supplied as a lyophilized powder for injection, which was dissolved in NS before use. After 2 weeks, all mice were anesthetized with sodium pentobarbital (50 mg/kg) by IP. Under anesthesia, blood samples were collected from the retro‐orbital venous plexus. After centrifugation at 1500 × *g* for 10 min, the supernatant serum was aliquoted and stored at −80°C. Subsequently, the mice were euthanized by IP overdose of sodium pentobarbital (200 mg/kg), and the heart tissue was quickly collected and rinsed with pre‐cooled PBS to remove residual blood. The major blood vessels and surrounding connective tissues were removed. For histopathological analysis, the intact heart was fixed, embedded in paraffin, and subjected to longitudinal sectioning. Histopathological examination and fibrosis evaluation were primarily conducted on the ventricular myocardial regions of the sections, with avoidance of atrial tissue, cardiac valves, major blood vessels, epicardial adipose tissue, and other nonmyocardial areas. For biochemical assays, reverse transcription‐quantitative polymerase chain reaction (RT‐qPCR), and WB analysis, ventricular myocardial tissue samples were harvested, rapidly snap‐frozen in liquid nitrogen, and stored at −80°C for subsequent analyses.

#### 2.3.2 Histopathological Analysis

Hematoxylin and eosin (H&E) staining and Masson's trichrome staining were used to assess histopathological alterations in mouse myocardial tissues. Fixed mouse hearts were subjected to gradient dehydration using a graded series of ethanol solutions (70%–100%), followed by xylene‐mediated clearing and paraffin embedding. The paraffin blocks were sectioned into 4 μm‐thick slices. Sections were dewaxed in xylene, followed by rehydration through a graded ethanol series, and then stained with H&E dye (Servicebio, G1076) and Masson's trichrome dye (Servicebio, G1006), respectively. The pathological morphology and myocardial fibrosis extent were mainly evaluated in the ventricular myocardial region under an optical microscope (Olympus BX51), and representative images were collected. The pathological scoring criteria were adopted in accordance with previously published studies (Al‐Amran and Shahkolahi 2014). This assay included six mice per group (*n* = 6 biological replicates).

#### 2.3.3 Cardiac Function Assessment

Echocardiography (ECHO) was performed using a desktop small animal ultrasound system (Vinno D650LAB, Vinno Technology, China). In brief, mice were anesthetized by 3% isoflurane and then positioned supine on a heated surgical platform. Anesthesia was maintained by continuous inhalation of 1.5% isoflurane. After the chest depilation was completed, the ultrasound coupling gel was applied evenly and the probe was placed at the left sternal border to obtain a clear parasternal long‐axis image of the left ventricle (LV). For each mouse, at least three consecutive, stable cardiac cycles of M‐mode images were recorded. Key systolic function parameters, including ejection fraction (EF) and fractional shortening (FS), were measured using the instrument's integrated analytical software. This assay included six mice per group (*n* = 6 biological replicates).

#### 2.3.4 Biochemical Analysis of Myocardial Tissue Homogenate and Serum

Following the manufacturer's instructions strictly, the levels of superoxide dismutase (SOD, Catalog: A001), malondialdehyde (MDA, Catalog: A003), glutathione peroxidase (GSH‐Px, Catalog: A005), catalase (CAT, Catalog: A007), IL‐1β (Catalog: H002‐1‐2), IL‐6 (Catalog: H007), and TNF‐α (Catalog: H051) in the ventricular myocardial tissue supernatants were measured using assay kits. Following the manufacturer's instructions strictly, the serum samples collected from mice in each group were assayed for the creatine kinase (CK, Catalog: A032), lactate dehydrogenase (LDH, Catalog: A020), cardiac troponin I (cTnI, Catalog: JL11280, Jianglai Biotechnology), and cTnT (Catalog: JL40538, Jianglai Biotechnology) levels using corresponding detection kits. Finally, absorbance readings for all biochemical parameters were measured at their respective wavelengths using an automated microplate reader (Molecular Devices, SpectraMax i3x), and the results were exported for analysis. This assay included 10 mice per group (*n* = 10 biological replicates).

#### 2.3.5 ROS Staining

Fresh frozen tissue sections were rewarmed at room temperature, and excess moisture was removed. Dihydroethidium (DHE, Sigma, D7008) staining solution was added, and the sections were incubated in a light‐protected constant‐temperature incubator at 37°C for 30 min. Subsequently, the sections were washed with PBS 3 times (5 min per wash), followed by the addition of DAPI staining (Servicebio, G1012) solution and a 10‐min light‐protected incubation. After another 3 washes with PBS (5 min per wash), an anti‐fluorescence quenching agent (Servicebio, G1401) was added before mounting the sections. Finally, images were captured under a fluorescence microscope (Nikon, Eclipse C1), and image quantification analysis was performed using ImageJ software. This assay included 6 mice per group (*n* = 6 biological replicates).

#### 2.3.6 TUNEL Staining

Following deparaffinization and rehydration, the tissue sections were treated with proteinase K to improve tissue permeability and washed 3 times (5 min each) in PBS. A sufficient volume of the TUNEL reaction mixture was prepared according to the kit instructions (Servicebio, G1504). The TUNEL reaction mixture was applied to the sections and incubated at 37°C for 1 h. After another series of PBS washes, the sections were counterstained with DAPI staining (Servicebio, G1012). Images were acquired using a Nikon ECLIPSE C1 fluorescence microscope and subjected to quantitative analysis using ImageJ software. This assay included six mice per group (*n* = 6 biological replicates).

#### 2.3.7 RT‐qPCR

Total RNA was isolated from the samples using TRIzol reagent (15596‐026, Invitrogen) following the manufacturer's protocol. The concentration and purity of the RNA were quantified using an ultramicro spectrophotometer (Denovix DS‐11). Total RNA was reverse‐transcribed into cDNA using a reverse transcription kit (CW2582M, Cwbiosciences, China). Quantitative real‐time PCR was performed using SYBR Green and the primers listed in Table 2. β‐actin was used as the internal reference gene, and the relative expression level of the target gene was calculated using the 2^−ΔΔCt^ method. This assay included three mice per group (*n* = 3 biological replicates).

**TABLE 2:** Sequences of the target gene primers.

Gene name	Primer F	bp	Primer R	bp
IL‐1β	CACCTCACAAGCAGAGCACAAG	22	GCATTAGAAACAGTCCAGCCCATAC	25
IL‐6	CACGCTCTTCTGTCTACTGAACTTC	25	CTTGGTGGTTTGTGAGTGTGAGG	23
TNF‐α	GAGACTTCCATCCAGTTGCCTTC	23	TGTTGGGAGTGGTATCCTCTGTG	23
β‐actin	TGGGAATGGGTCAGAAGGA	19	ATTGAGAAAGGGCGTGGC	18

### 2.4 Cell Experiment Validation

#### 2.4.1 Cell Culture

H9c2 cells (CL‐0089, ProCell, China) were cultured in high‐glucose Dulbecco's modified Eagle medium (DMEM) supplemented with 10% fetal bovine serum (FBS) and 1% penicillin/streptomycin (P/S) solution. AC16 human cardiomyocytes (CL‐0790, ProCell, China) were cultured in DMEM/F12 supplemented with 10% FBS and 1% P/S solution. Both cell lines were cultured under standard conditions in a humidified incubator maintained at 37°C with 5% CO_2_. Upon reaching 80%–90% confluence, the cells were trypsinized with 0.25% trypsin–EDTA solution for passaging. Cells in the logarithmic growth phase with high viability were selected for subsequent drug intervention experiments. Furthermore, H9c2 cells were treated with varying concentrations of ATO (2.5, 5, 10, 20, 40 μM) and 10Gin (2.5, 5, 10, 20, 40 μM) for 24 h, and the optimal administration concentrations of ATO and 10Gin were determined based on the results of the cell counting kit‐8 (CCK‐8) assay. This assay included 6 biological replicates (*n* = 6).

#### 2.4.2 Cell Treatment

Based on the CCK‐8 assay results, the exact cell viability data for different concentrations of ATO and 10Gin are provided in Supporting Information 1 (Table S3). ATO reduced H9c2 cell viability in a concentration‐dependent manner. An in vitro cell injury model was established by treating H9c2 cells with 10 μM ATO for 24 h, a dosing regimen also consistent with that reported in previous peer‐reviewed studies (Zhang et al. 2017). 5 μM and 10 μM 10Gin were designated as the low‐dose and high‐dose groups for subsequent cell experiments, respectively. H9c2 cells in the logarithmic growth phase were randomly assigned to five groups: (1) CON group in cell experiments (C‐CON); (2) ATO group in cell experiments (C‐ATO); (3) Low‐dose 10Gin + ATO co‐treated group in cell experiments (C‐L‐10Gin); (4) High‐dose 10Gin + ATO co‐treated group in cell experiments (C‐H‐10Gin); (5) Antagonist LY294002 pretreatment 1 h, then high‐dose 10Gin + ATO co‐treated group in cell experiments (C‐H‐10Gin + LY294002). Briefly, the C‐CON group was cultured in complete medium supplemented with an equal volume of DMSO (final concentration ≤ 0.1%) for 24 h. The C‐ATO group was treated with 10 μM ATO for 24 h. The C‐L‐10Gin and C‐H‐10Gin intervention groups were co‐treated with 10 μM ATO plus 5 μM or 10 μM 10Gin for 24 h, respectively. The pathway reversal verification group (C‐H‐10Gin + LY294002) was pre‐incubated with 20 μM LY294002 for 1 h, then co‐treated with 10 μM ATO and 10 μM 10Gin for another 24 h (Wang et al. 2021).

To further validate the cardioprotection of 10Gin in a human cardiomyocyte cell line, AC16 cells were used for additional experiments. Based on the effective concentration identified in H9c2 cells, high‐dose 10Gin (10 μM) was selected for AC16 cell validation. AC16 cells were divided into four groups: AC16‐CON, AC16‐ATO, AC16‐10Gin, and AC16‐10Gin + LY294002. The AC16‐CON group was cultured in complete medium containing an equal volume of vehicle. The AC16‐ATO group was treated with 10 μM ATO for 24 h. The AC16‐10Gin group was co‐treated with 10 μM ATO and 10 μM 10Gin for 24 h. The AC16‐10Gin + LY294002 group was pretreated with 20 μM LY294002 for 1 h, followed by co‐treatment with 10 μM ATO and 10 μM 10Gin for another 24 h. All cells were cultured in a humidified incubator with 5% CO_2_ at 37°C.

#### 2.4.3 Cell Viability Assay

For H9c2 cells, cell viability was evaluated using the CCK‐8 assay. H9c2 cells in the logarithmic growth phase were seeded into 96‐well plates. Following cell adhesion, the cells were treated with drugs according to the groups. After completion of the intervention, 10% volume of CCK‐8 was added to each well, followed by incubation in the dark at 37°C for 2 h. The absorbance at 450 nm was measured using a microplate reader, and the relative cell viability was subsequently calculated. This assay included six biological replicates (*n* = 6). For AC16 cells, cell viability was assessed by Calcein‐AM/PI staining (E‐CK‐A354, Elabscience, China). After drug treatment, AC16 cells were collected, washed with PBS, and stained according to the manufacturer's instructions. The percentages of live and dead cells were detected by flow cytometry, and relative cell viability was calculated. This assay included three biological replicates (*n* = 3).

#### 2.4.4 Biochemical Analyses in Cell Experiments

For H9c2 cells, after completion of the cell intervention, the culture supernatants and cell pellets from each experimental group were collected. Following centrifugation of the supernatant to remove impurities, the levels of CK, LDH, cTnT, cTnI, IL‐1β, IL‐6, and TNF‐α were measured using corresponding commercial kits. The cell pellets were washed with PBS, lysed, and centrifuged to obtain the cell lysates. The intracellular levels of SOD, MDA, and GSH‐Px were measured using the corresponding commercial assay kits. All procedures were performed strictly in accordance with the manufacturer's instructions provided with the commercial assay kits. For AC16 cells, the levels of CK, LDH, and cTnI in the culture supernatants were measured to evaluate ATO‐induced HI and the protection of 10Gin. All procedures were performed strictly according to the manufacturers' instructions. These assays included 6 biological replicates (*n* = 6). The absorbance values at the corresponding wavelengths were measured with a microplate reader, and the levels of each index were calculated.

#### 2.4.5 Cellular ROS Fluorescence Staining

The ROS levels in each group were detected by incubating H9c2 cells with 10 μM DCFH‐DA (D6470, Solarbio, China) fluorescent probe at 37°C in the dark for 30 min. After staining, the samples were washed twice with PBS. Fluorescence images were captured using a fluorescence microscope, and the fluorescence intensity was quantitatively analyzed using the ImageJ software. Six biological replicates were included in this assay (*n* = 6).

#### 2.4.6 Flow Cytometric Analysis

Flow cytometry was used to assess apoptosis and intracellular ROS levels in H9c2 and AC16 cells. The apoptosis levels of cells in each group were evaluated by staining with an Annexin V‐FITC/PI apoptosis detection kit (AP101, Lianke Biology, China), while the ROS detection was performed using the DCFH‐DA probe. The detailed experimental procedures are described in Supporting Information 1 (page 2). For H9c2 cells, these assays included six biological replicates (*n* = 6). For AC16 cells, these assays included three biological replicates (*n* = 3).

### 2.5 Western Blotting

Total proteins were isolated from heart tissue, H9c2 cells, and AC16 cells using RIPA lysis buffer, and protein concentrations were detected using the BCA protein assay kit (G2026, Servicebio, China). Subsequently, equal amounts of protein lysates were resolved by 10% SDS‐PAGE and transferred onto PVDF membranes. After blocking with 5% nonfat milk for 1 h, the membranes were incubated overnight at 4°C with primary antibodies. Following washing, membranes were incubated for 1 h at room temperature with HRP‐conjugated goat anti‐rabbit IgG and goat anti‐mouse IgG secondary antibodies. The dilution ratios of all antibodies were provided in Supporting Information 1 (Table S2, page 1). Immunoreactive bands were visualized using ECL reagents and quantified by densitometry. This assay included three biological replicates (*n* = 3).

### 2.6 Statistical Analysis

Unless otherwise specified, data are presented as the mean ± standard error of the mean (SEM). Group comparisons were performed using one‐way ANOVA with Tukey's post hoc test (Origin 2021, OriginLab Inc.). A *p*‐value of less than 0.05 was considered statistically significant.

## 3 Results

### 3.1 Screening 10Gin Potential Targets in Ameliorating ATO‐Induced HI

**FIGURE 1:**
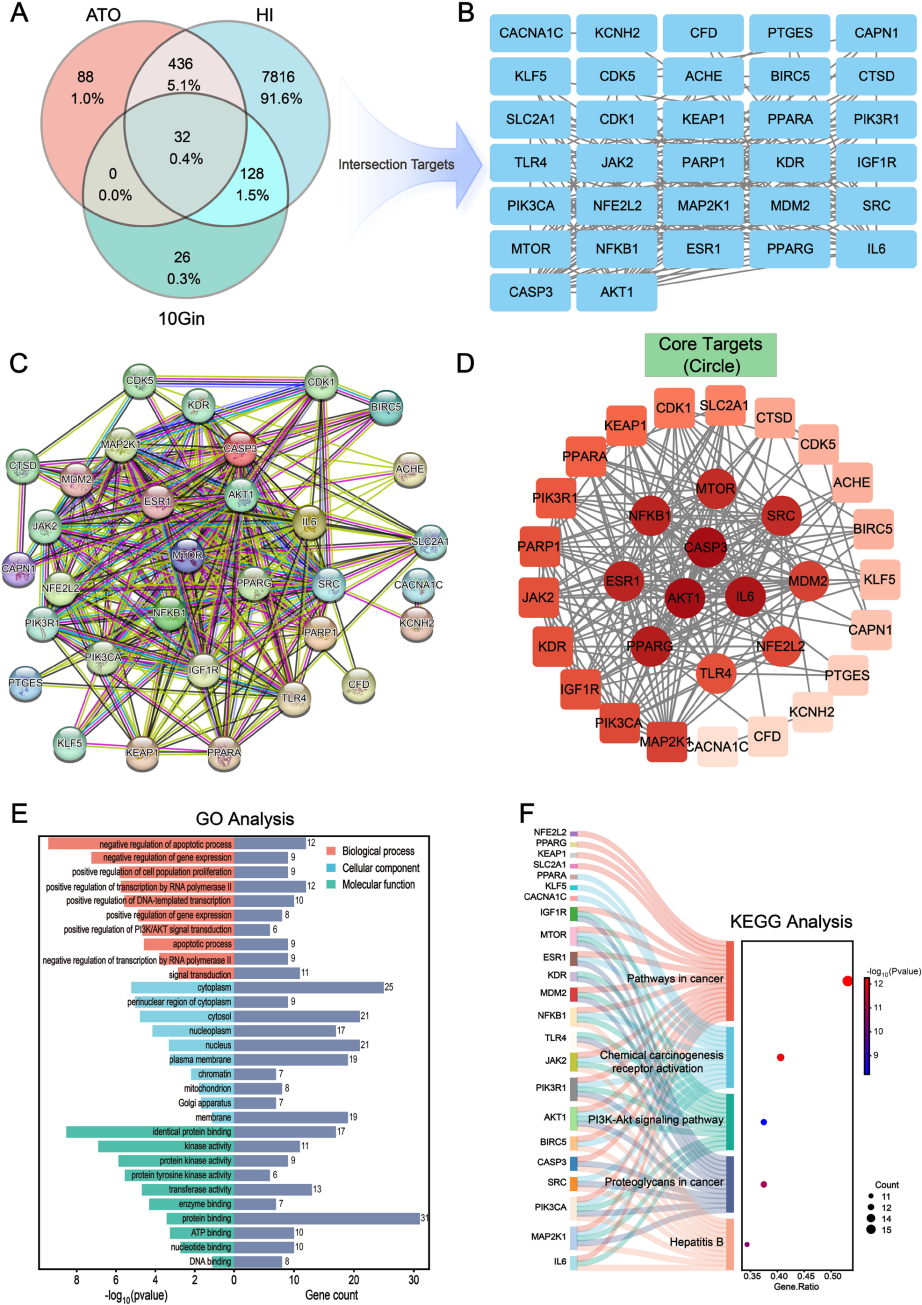
NP analysis of 10Gin against ATO‐induced HI. (A) Venn diagram of 10Gin, ATO, and HI targets. (B) Intersection targets of 10Gin, ATO, and HI. (C) Visualization of PPI network construction. (D) Visualization of core intersecting targets (sorted by DC value, with darker red indicating higher DC value). (E) GO enrichment analysis. (F) Sankey diagram of KEGG pathway enrichment analysis (TOP 5 pathways).

As shown in Figure 1, 186 unique targets associated with 10Gin were identified using the TCMSP, STP, and SPTP databases. From the CTD database, 556 targets related to ATO were collected. Targets associated with HI were retrieved from OMIM, GeneCards, and DrugBank, yielding 8412 unique targets after deduplication. Venn diagram analysis was performed to identify the intersection among the targets of 10Gin, ATO, and HI (Figure 1A). This analysis revealed 32 potential therapeutic targets through which 10Gin may ameliorate ATO‐induced HI (Figure 1A). These 32 overlapping targets (Figure 1B), including AKT1, IL6, CASP3, PPARG, ESR1, TLR4, and NFKB1, were associated with the regulation of cardiomyocyte apoptosis, inflammatory response, oxidative stress, and survival signaling pathways.

### 3.2 PPI Network Construction and Core Target Screening

The STRING database, a repository integrating PPI from public databases and literature, plays a pivotal role in PPI network studies (Szklarczyk et al. 2025). Beyond visualizing interaction networks, it provides annotations for protein families, biological pathways, and subcellular localization (Szklarczyk et al. 2025). As shown in Figure 1C, we imported the 32 intersecting targets identified previously into STRING to establish the PPI network. The PPI network contained 32 nodes and 230 edges, with no isolated nodes. To identify core targets, topological analysis was performed using Cytoscape 3.9.1 with screening criteria set at values exceeding the median of three centrality metrics (DC > 17.00, BC > 4.09, CCT > 0.67). The resulting core targets were visualized and ranked by DC values (Figure 1D). Targets with higher DC values exhibited greater connectivity within the PPI network.

The top five ranked core targets were AKT1, CASP3, IL6, PPARG, and ESR1, which are also the mediators of ATO‐induced HI. Studies have shown that ATO triggers cardiomyocyte apoptosis by activating the pro‐apoptotic executioner protein CASP3 (Rao et al. 2023), induces myocardial inflammatory cascades via upregulation of the core pro‐inflammatory cytokine IL6 (Qin et al. 2024), disrupts cardiomyocyte survival homeostasis through inhibition of the serine/threonine kinase AKT1 (Thangapandiyan et al. 2024), and exacerbates myocardial oxidative stress by downregulating the nuclear receptor PPARG (Li, Ma, et al. 2022). Furthermore, studies have shown that inhibiting ESR1 can exacerbate HI (Chen et al. 2025). NP analysis in the present study predicted that these five core targets served as key regulatory nodes through which 10Gin antagonizes ATO‐induced cardiotoxicity and are closely associated with multiple critical signaling pathways involved in HI.

### 3.3 GO and KEGG Enrichment Analyses

To further elaborate on the potential biological functions and signaling pathways associated with these 32 intersecting targets, we conducted GO and KEGG enrichment analyses via the DAVID database (Sherman et al. 2024). Setting a significance threshold of *p* < 0.01, we identified 109 KEGG pathways, 84 BP terms, 15 CC terms, and 27 MF terms in total. In the BP category, the most significantly enriched terms primarily included negative regulation of apoptotic process, negative regulation of gene expression, regulation of cell population proliferation, positive regulation of RNA polymerase II‐mediated transcription, and apoptotic process. In the MF category, the most significantly enriched terms included protein binding, identical protein binding, transferase activity, protein serine/threonine kinase activity, and ATP binding. In the CC category, the targets were predominantly enriched in the cytoplasm, nucleus, and plasma membrane, which was consistent with the subcellular localization of the five core targets and aligned with the spatial distribution characteristics of intracellular signal transduction in cardiomyocytes under ATO exposure (Figure 1E).

The KEGG enrichment analysis results indicated that the overlapping targets were significantly enriched in the PI3K/AKT signaling pathway, cancer pathways, chemical carcinogenesis‐receptor activation, proteoglycans in cancer, and hepatitis B‐related pathways (Figure 1F). Among these enriched pathways, the PI3K/AKT signaling pathway was the most significantly enriched cardiovascular‐related regulatory pathway, which is highly associated with the top‐ranked core target AKT1. In conclusion, the NP analysis in this study predicted that 10Gin may alleviate ATO‐induced cardiotoxicity primarily by targeting five core targets (AKT1, CASP3, IL6, PPARG, and ESR1) and modulating the PI3K/AKT signaling pathway as well as its downstream BPs, including apoptosis, inflammation, and oxidative stress.

### 3.4 10Gin Ameliorates the General Observations of ATO‐Treated Mice

As shown in Table 1, the initial body weight, final body weight, average food intake, and average water intake of mice in each group were adopted as indicators for the general observation of the mice. The results showed that, in comparison with the CON group, mice in the ATO group had a significant reduction in body weight, food intake, and water intake (*p* < 0.01). Following 10Gin treatment, mice in the H‐10Gin group showed significantly increased body weight, food consumption, and water consumption (*p* < 0.05 or *p* < 0.01). In addition, no significant differences were observed between the CON and A‐10Gin groups.

### 3.5 10Gin Attenuates Myocardial Histopathological Damage and Serum Injury Biomarkers in Mice

**FIGURE 2:**
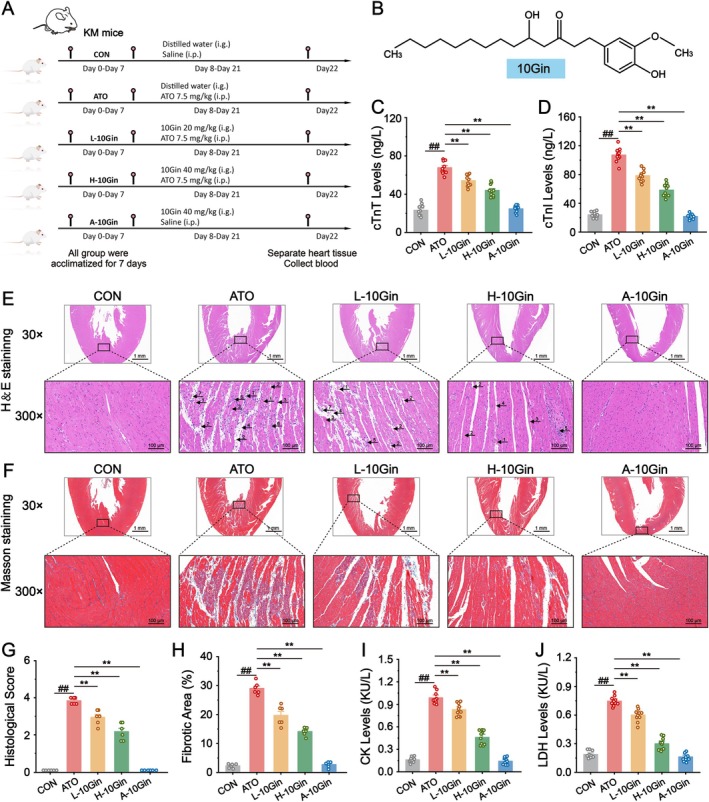
10Gin alleviates ATO‐induced myocardial histopathological changes and reduces myocardial injury biomarker levels in mice. (A) Experimental operations and processes. (B) Chemical structure of 10Gin. (C, D) Quantitative analysis of serum cTnT and cTnI levels (*n* = 10). (E, F) Representative H&E and Masson staining images in each group (30×, scale bar = 1 mm; 300×, scale bar = 100 μm) (The numbered arrows in the H&E staining images indicate the following pathological features: 1. myocardial interstitial congestion; 2. myocardial cell vacuolar degeneration; 3. eosinophilic enhancement, color deepening; 4. myocardial interstitial edema; 5. myocardial inflammatory cell infiltration; 6. karyorrhexis; 7. myocardial interstitial inflammatory cell infiltration). (G) Quantitative analysis of H&E staining histological scores (*n* = 6). (H) Quantitative analysis of cardiac fibrosis area (*n* = 6). (I, J) The levels of serum CK and LDH (*n* = 10). Data are represented as mean ± SEM. ^##^ *p* < 0.01 vs. CON; **p < 0.01 vs. ATO.

As demonstrated in Figure 2, H&E staining of myocardial tissue revealed well‐preserved histoarchitecture in both the CON and A‐10Gin groups, characterized by tightly aligned cardiac fibers with distinct cross‐striations, uniformly eosinophilic cytoplasmic staining, centrally positioned oval nuclei with homogeneous basophilic staining, clearly delineated capillary networks without interstitial edema or exudates, and absence of inflammatory infiltrates or necrotic foci. In contrast, the ATO group exhibited marked pathological alterations in myocardial tissue, including myocardial interstitial congestion, cardiomyocyte vacuolar degeneration, increased cytoplasmic eosinophilia, myocardial interstitial edema, myocarditis cell infiltration, karyorrhexis, and myocardial interstitial inflammatory cell infiltration (*p* < 0.01). After treatment with 10Gin, both the L‐10Gin and H‐10Gin groups exhibited differential improvements in the scores of myocardial histopathological changes (*p* < 0.01). Masson's trichrome staining corroborated the H&E findings, revealing well‐preserved myocardial architecture with minimal collagen deposition in the CON and A‐10Gin groups. In contrast, the ATO group exhibited extensive fibrotic networks accompanied by cellular swelling and interstitial edema (*p* < 0.01). Both L‐10Gin and H‐10Gin interventions attenuated fibrosis, reduced collagen deposition, and ameliorated histopathological alterations in myocardial tissue (*p* < 0.01). Furthermore, 10Gin's cardioprotection against ATO‐induced HI was further validated by the alterations in serum cardiac injury biomarkers. In comparison with the CON group, ATO treatment significantly increased the levels of CK, LDH, cTnT, and cTnI (*p* < 0.01). In contrast, 10Gin treatment significantly suppressed these elevations (*p* < 0.01).

### 3.6 10Gin Ameliorates ATO‐Induced Cardiac Dysfunction of Mice

**FIGURE 3:**
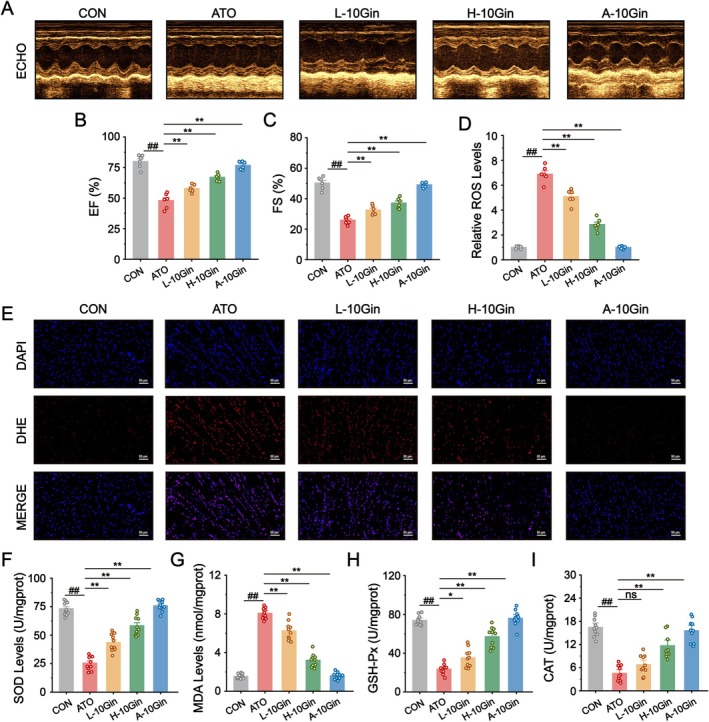
10Gin improves cardiac function and attenuates oxidative stress levels in ATO‐induced HI mice. (A) Representative ECHO images of each group. (B, C) Quantitative analysis of EF and FS levels (*n* = 6). (D, E) Representative DHE staining images and quantitative analysis of their relative fluorescence intensity (400×, scale bar = 50 μm) (*n* = 6). (F–I) Quantitative measurement of SOD, MDA, GSH‐Px, and CAT (*n* = 10). Data are represented as mean ± SEM. ^##^ *p* < 0.01 vs. CON; ns, no significance, *p < 0.05, **p < 0.01 vs. ATO.

Cardiac systolic function was evaluated by ECHO, with EF and FS serving as the primary assessment indices. As shown in Figure 3A–C, compared with the CON group, the ATO treatment significantly reduced EF and FS levels (*p* < 0.01), suggesting obvious LV systolic dysfunction. Conversely, 10Gin treatment significantly elevated the levels of EF and FS, thereby ameliorating cardiac dysfunction (*p* < 0.01). Furthermore, it is noteworthy that no significant differences in EF and FS were observed between the CON group and the A‐10Gin group, indicating that high‐dose 10Gin alone did not impair the baseline cardiac function of normal mice under the present experimental conditions. These ECHO findings further supported that 10Gin can alleviate ATO‐induced cardiac dysfunction and exert protective effects on myocardial contractile function.

### 3.7 10Gin Mitigates Oxidative Stress in Mouse Myocardial Tissue

As shown in Figure 3D–I, in comparison with the CON group, the ATO group exacerbated oxidative stress injury in myocardial tissue. This was evidenced by significantly increased levels of ROS and MDA (*p* < 0.01), along with significantly decreased activities of the antioxidant enzymes SOD, GSH‐Px, and CAT within the tissue (*p* < 0.01). Following 10Gin treatment, ROS and MDA levels in the L‐10Gin and H‐10Gin groups were significantly decreased, whereas the antioxidant enzyme activities were remarkably increased (*p* < 0.05 or *p* < 0.01). This coordinated restoration of redox homeostasis indicated that 10Gin ameliorates ATO‐induced HI through augmentation of endogenous antioxidant defenses.

### 3.8 10Gin Mitigates Inflammatory Cytokine Levels in Mouse Myocardium

**FIGURE 4:**
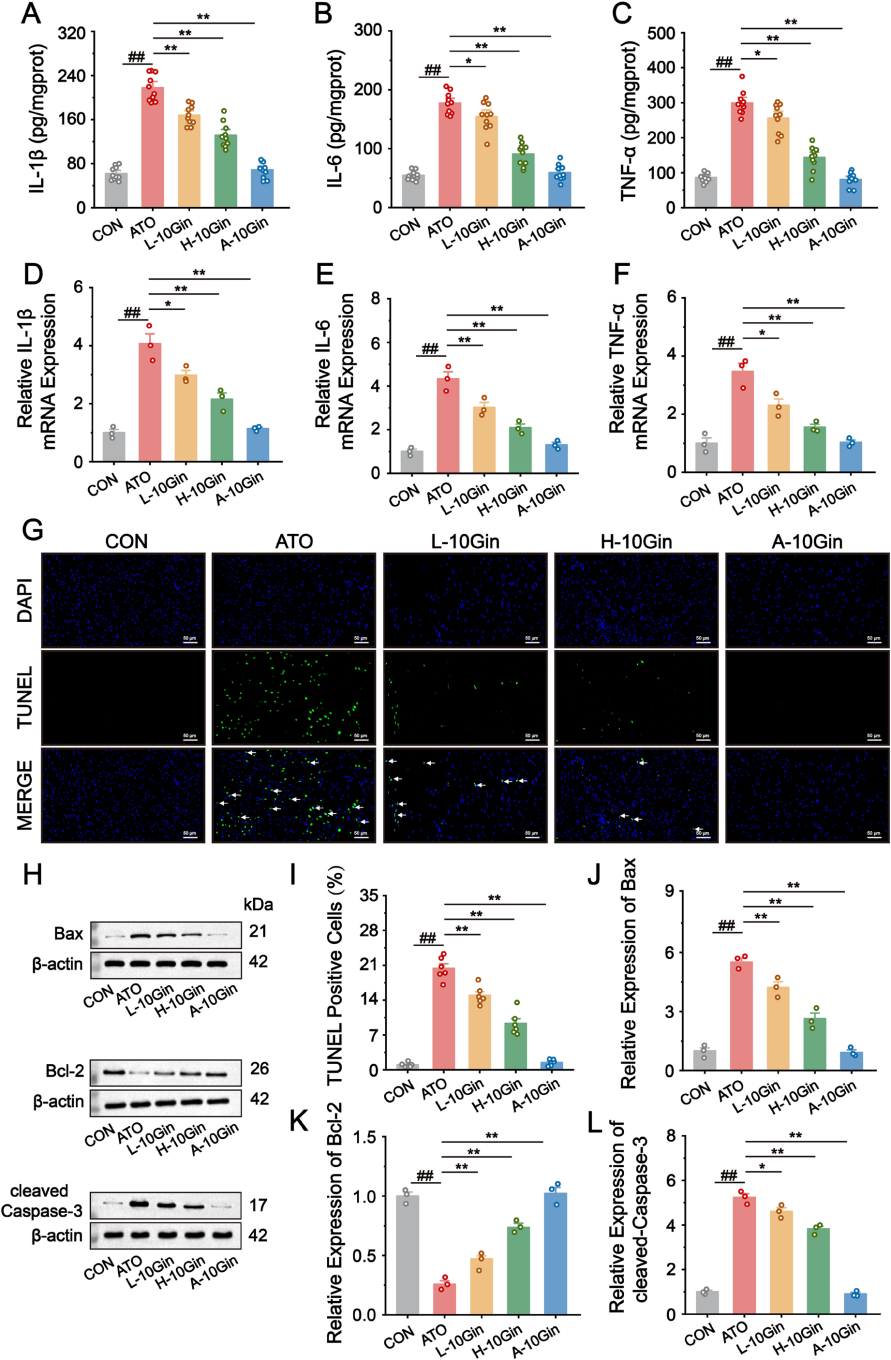
10Gin alleviates myocardial inflammatory responses and apoptosis in ATO‐induced HI mice. (A–C) Quantitative measurement of IL‐1β, IL‐6, and TNF‐α levels (*n* = 10). (D–F) Quantitative analysis of relative mRNA expression levels of IL‐1β, IL‐6, and TNF‐α (*n* = 3). (G) Representative TUNEL staining images of each group (400×, scale bar = 50 μm). (H) Representative WB bands of Bax, Bcl‐2, and cleaved Caspase‐3. (I) Quantitative analysis of TUNEL staining (*n* = 6). (J–L) The protein expressions of Bax, Bcl‐2, and cleaved Caspase‐3 (*n* = 3). Data are represented as mean ± SEM. ^##^ *p* < 0.01 vs. CON; *p < 0.05, **p < 0.01 vs. ATO.

The results showed that compared with the CON group, the levels of pro‐inflammatory cytokines IL‐1β, IL‐6, and TNF‐α were significantly increased (*p* < 0.01), indicating that ATO induced a robust inflammatory response. 10Gin treatment significantly reduced the levels of inflammatory factors, exerting anti‐inflammatory effects (*p* < 0.05 or *p* < 0.01) (Figure 4A–C). Furthermore, RT‐qPCR was used to further validate the anti‐inflammatory effect of 10Gin. The results demonstrated that the mRNA expression of the aforementioned inflammatory factors in the myocardial tissue of the ATO group was also significantly upregulated (*p* < 0.01). 10Gin treatments significantly downregulated the mRNA levels of these inflammatory factors, inhibiting the inflammatory response at the transcriptional level (*p* < 0.05 or *p* < 0.01) (Figure 4D–F). These results suggested that 10Gin exerted cardioprotective effects by enhancing anti‐inflammatory capacity.

### 3.9 10Gin Suppresses Cardiomyocyte Apoptosis in Mice

As shown in Figure 4G,I, in comparison with the CON group, the ATO group markedly promoted apoptosis, as evidenced by a significantly increased percentage of TUNEL‐positive cells of myocardial tissue (*p* < 0.01). By contrast, relative to the ATO group, both the L‐10Gin and H‐10Gin groups brought about a significant reduction in TUNEL‐positive cells (*p* < 0.01). Furthermore, changes in the expression levels of apoptosis‐related proteins further confirmed these findings (Figure 4H,J–L). Compared to the CON group, the ATO group markedly upregulated the protein expressions of Bax and cleaved Caspase‐3, while significantly decreasing the expression levels of Bcl‐2 (*p* < 0.01). Following 10Gin treatment, both the L‐10Gin and H‐10Gin groups markedly decreased the protein expressions of Bax and cleaved Caspase‐3 and significantly increased the expression levels of Bcl‐2 (*p* < 0.05 or *p* < 0.01). These results collectively suggested that 10Gin attenuates ATO toxicity‐induced HI via anti‐apoptotic effects.

### 3.10 10Gin Activates PI3K/AKT Pathway via Phosphorylation in Myocardium of Mice

**FIGURE 5:**
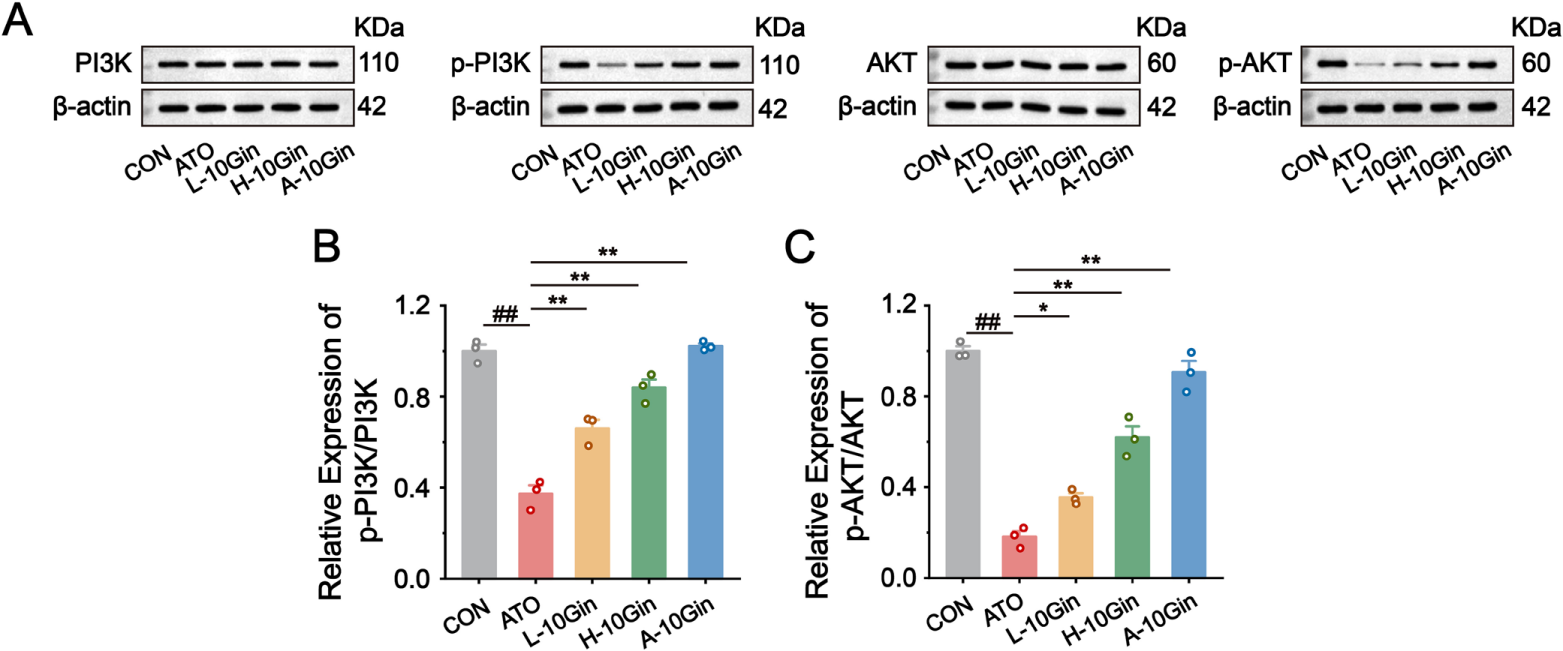
10Gin attenuates ATO‐induced HI through activation of the PI3K/AKT pathway. (A–C) Representative WB bands and the relative expression ratio of p‐PI3K/PI3K and p‐AKT/AKT (*n* = 3). Data are represented as mean ± SEM. ^##^ *p* < 0.01 vs. CON; *p < 0.05, **p < 0.01 vs. ATO.

As shown in Figure 5, compared to the CON group, ATO treatment significantly downregulated the relative expression ratio of p‐PI3K/PI3K and p‐AKT/AKT (*p* < 0.01). Treatment with 10Gin significantly increased both ratios of p‐PI3K/PI3K and p‐AKT/AKT compared with the ATO group (*p* < 0.05 or *p* < 0.01). These findings indicated that 10Gin may ameliorate ATO toxicity‐induced HI by promoting the phosphorylation of PI3K and AKT, thereby activating this signaling pathway.

### 3.11 H9c2 Cell Validation of the Cardioprotective Effect of 10Gin

#### 3.11.1 10Gin Attenuates ATO‐Induced Viability Reduction and Injury in H9c2 Cells

**FIGURE 6:**
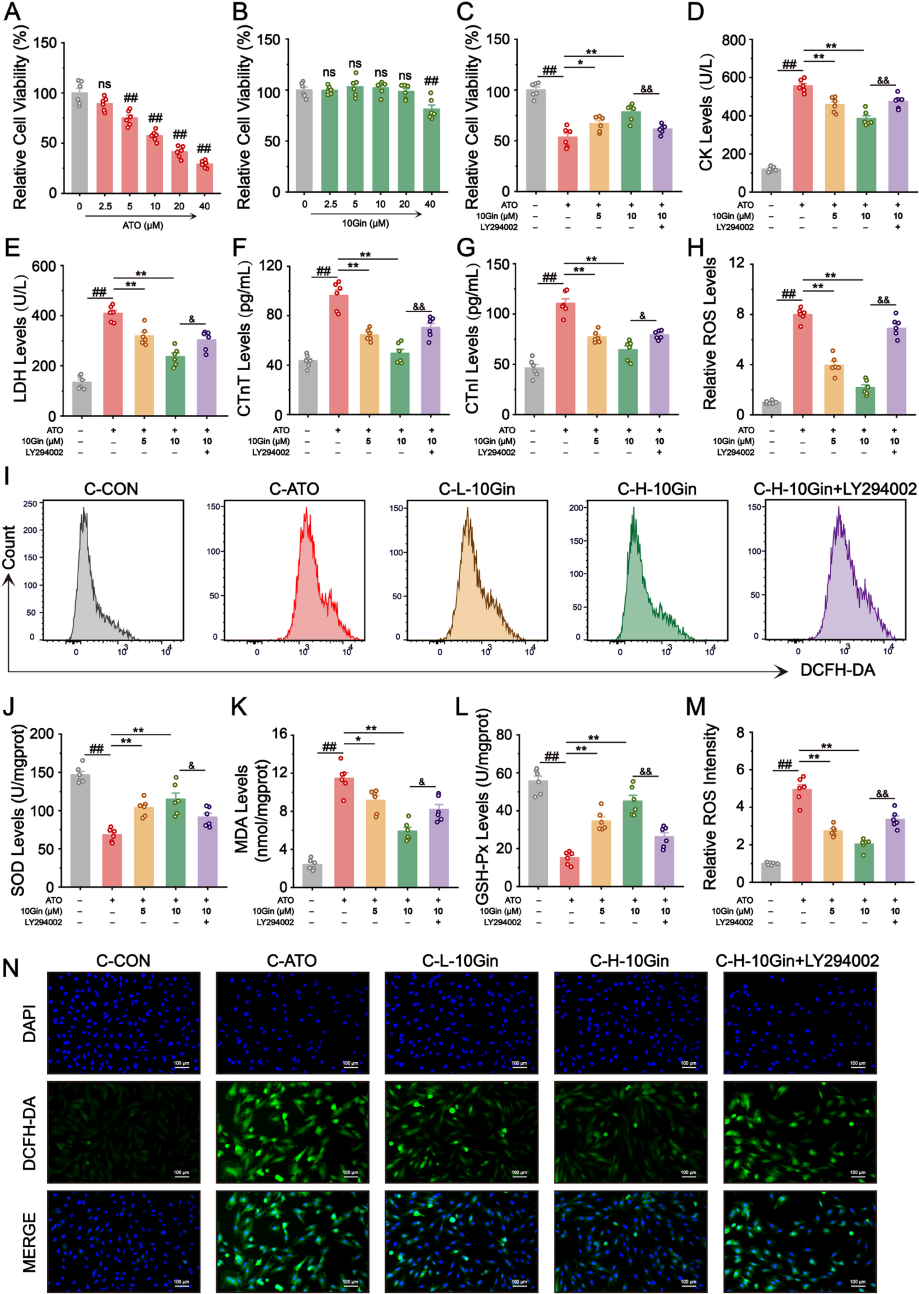
10Gin attenuates ATO‐induced H9c2 cell injury by enhancing cell viability, reducing myocardial biomarkers, and mitigating oxidative stress levels. (A, B) Different concentrations of ATO (2.5–40 μM) and 10Gin (2.5–40 μM) on the viability of H9c2 cells (*n* = 6). (C) Effects of 10Gin and LY294002 on the viability of ATO‐injured H9c2 cells (*n* = 6). (D–G) Cell injury biomarkers CK, LDH, cTnT, and cTnI levels in the cell supernatant (*n* = 6). (H, I) Representative flow cytometry images of ROS and relative ROS levels (*n* = 6). (J–L) Intracellular SOD and GSH‐Px activities, and MDA content (*n* = 6). (M, N) Representative DCFH‐DA staining images and relative ROS intensity (200×, scale bar = 100 μm) (*n* = 6). Data are represented as mean ± SEM. ns, no significance, ^##^ *p* < 0.01 vs. C‐CON; *p < 0.05, **p < 0.01 vs. C‐ATO; ^&^ *p* < 0.05, ^&&^ *p* < 0.01 vs. C‐H‐10Gin.

To verify the regulatory role of the PI3K/AKT signaling pathway, as predicted by NP analysis, in the antagonism of ATO‐induced HI by 10Gin, the present study established an ATO‐injured H9c2 cell model and systematically verified the cardioprotective effects of 10Gin as well as its regulatory role in the PI3K/AKT pathway. As shown in Figure 6A,B, the cytotoxic effects of ATO and 10Gin on H9c2 cells were detected by CCK‐8 assay. The results demonstrated that ATO treatment reduced the relative viability of H9c2 cells in a concentration‐dependent manner (*p* < 0.01). Notably, 40 μM 10Gin significantly reduced the viability of H9c2 cells (*p* < 0.01). Based on the CCK‐8 assay results, 10 μM ATO was applied to H9c2 cells for 24 h to establish an in vitro cell injury model, a dosing regimen also consistent with that reported in previous peer‐reviewed studies (Zhang et al. 2017). 5 μM and 10 μM 10Gin were designated as the low‐dose and high‐dose groups for subsequent cell experiments, respectively.

As shown in Figure 6C, compared to the C‐CON group, the C‐ATO group significantly decreased the viability of H9c2 cells (*p* < 0.01). 10Gin intervention significantly enhanced the cell viability (*p* < 0.05 or *p* < 0.01). Notably, the addition of LY294002, an inhibitor of PI3K, partially reversed this effect relative to the C‐H‐10Gin group (*p* < 0.01), indicating that 10Gin can enhance cell viability and exert cardioprotective effects by modulating the PI3K/AKT pathway. Consistent with the cell viability findings, ATO treatment significantly elevated the levels of myocardial injury markers in the cell supernatant, including CK, LDH, cTnT, and cTnI (*p* < 0.01). Intervention with 10Gin significantly reduced the levels of these injury markers (*p* < 0.01) (Figure 6D–G). However, relative to the C‐H‐10Gin group, the addition of LY294002 partially attenuated the inhibitory effect of 10Gin on ATO‐induced elevation of myocardial injury markers (*p* < 0.05 or *p* < 0.01).

#### 3.11.2 10Gin Inhibits ATO‐Induced Oxidative Stress in H9c2 Cells

As shown in Figure 6H–N, flow cytometry, DCFH‐DA staining, and intracellular oxidative stress biochemical assays collectively demonstrated that the C‐ATO group significantly elevated oxidative stress levels (*p* < 0.01). In contrast, 10Gin intervention markedly suppressed ATO‐induced excessive ROS production and attenuated oxidative stress (*p* < 0.05 or *p* < 0.01). Critically, the addition of LY294002 partially attenuated the antioxidant effect of 10Gin, as evidenced by the elevation of ROS and MDA levels as well as the reduction of SOD and GSH‐Px activities (*p* < 0.05 or *p* < 0.01).

#### 3.11.3 10Gin Ameliorates ATO‐Induced Inflammation and Apoptosis in H9c2 Cells

**FIGURE 7:**
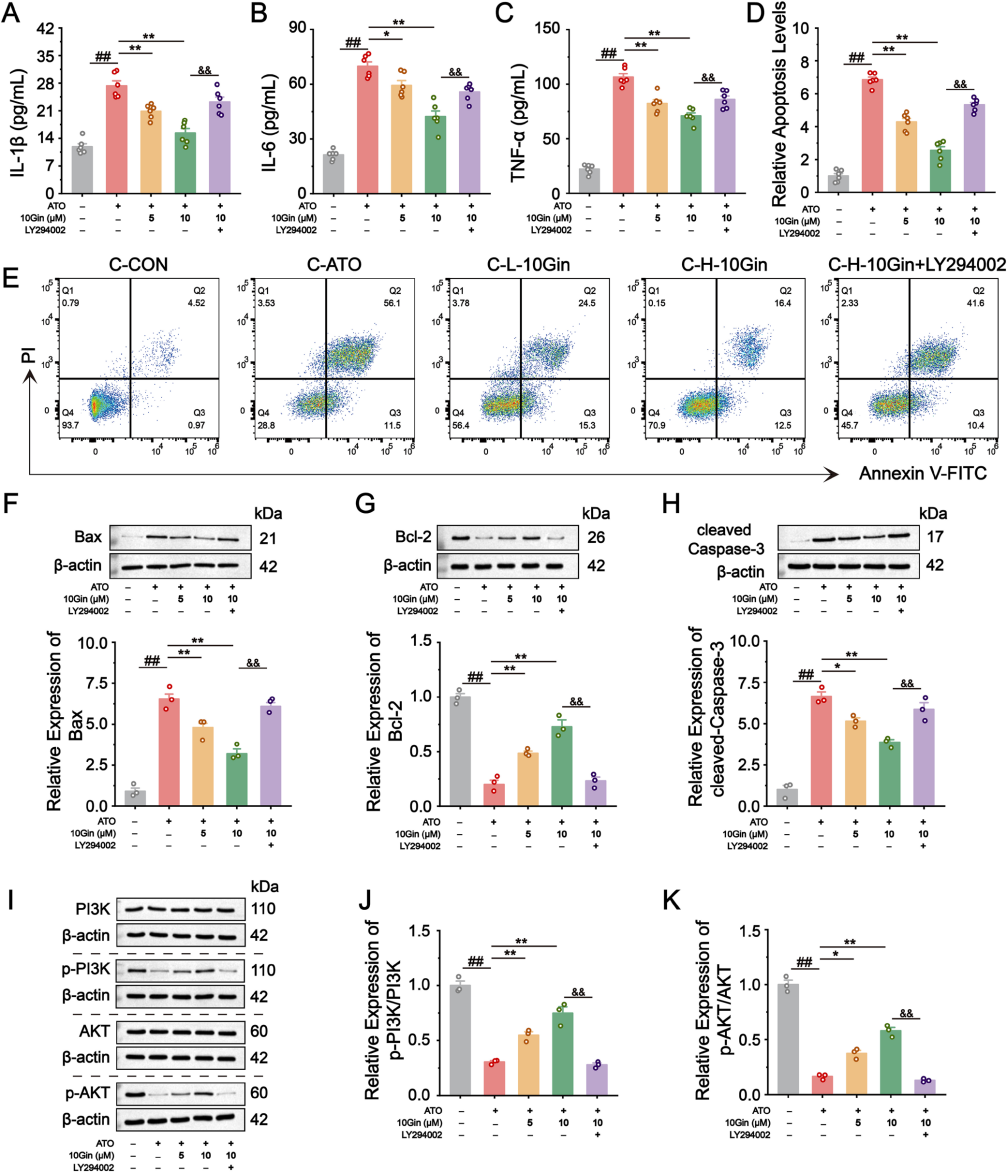
10Gin protects H9c2 cells against ATO‐induced injury through activation of the PI3K/AKT signaling pathway. (A–C) Levels of IL‐1β, IL‐6, and TNF‐α in the cell supernatant (*n* = 6). (D, E) Representative flow cytometry images of apoptosis and relative apoptosis levels (*n* = 6). (F–H) Representative WB bands and expression levels of Bax, Bcl‐2, and cleaved Caspase‐3 (*n* = 3). (I–K) Representative WB bands and the relative expression ratio of p‐PI3K/PI3K and p‐AKT/AKT (*n* = 3). Data are represented as mean ± SEM. ^##^ *p* < 0.01 vs. C‐CON; *p < 0.05, **p < 0.01 vs. C‐ATO; ^&&^ *p* < 0.01 vs. C‐H‐10Gin.

As shown in Figure 7A–C, ATO treatment significantly upregulated the levels of pro‐inflammatory cytokines, including IL‐1β, IL‐6, and TNF‐α in the cell supernatant (*p* < 0.01). Intervention with 10Gin markedly suppressed ATO‐induced upregulation of these pro‐inflammatory cytokines (*p* < 0.05 or *p* < 0.01). However, treatment with LY294002 partially reversed the anti‐inflammatory effect of 10Gin, as evidenced by a significant elevation in pro‐inflammatory cytokine levels relative to the C‐H‐10Gin group (*p* < 0.01). Consistent with the aforementioned findings, flow cytometry analysis revealed that 10Gin intervention significantly attenuated ATO‐induced cell apoptosis (*p* < 0.01) (Figure 7D,E). WB assays further validated the anti‐apoptotic effect of 10Gin, as 10Gin treatment markedly reversed the aberrant expression of apoptosis‐related proteins triggered by ATO (*p* < 0.05 or *p* < 0.01) (Figure 7F–H). In contrast, LY294002 pretreatment partially weakened the anti‐apoptotic effect of 10Gin, as evidenced by a significant increase in the cell apoptosis rate and pro‐apoptotic protein expression, accompanied by a decrease in anti‐apoptotic protein expression (*p* < 0.01).

#### 3.11.4 10Gin Exerts Cardioprotection by Activating the PI3K/AKT Signaling Pathway

As shown in Figure 7I–K, ATO treatment significantly downregulated the p‐PI3K/PI3K and p‐AKT/AKT ratios in H9c2 cells (*p* < 0.01). In contrast, treatment with 10Gin markedly upregulated the p‐PI3K/PI3K and p‐AKT/AKT ratios (*p* < 0.05 or *p* < 0.01). Consistent with our hypothesis, the PI3K inhibitor LY294002 pretreatment partially attenuated the 10Gin‐induced upregulation of PI3K and AKT phosphorylation levels (*p* < 0.01). These findings suggest that 10Gin may exert protective effects against ATO‐induced HI by activating the PI3K/AKT signaling pathway.

### 3.12 AC16 Cell Validation of the Cardioprotective Effect of 10Gin

**FIGURE 8:**
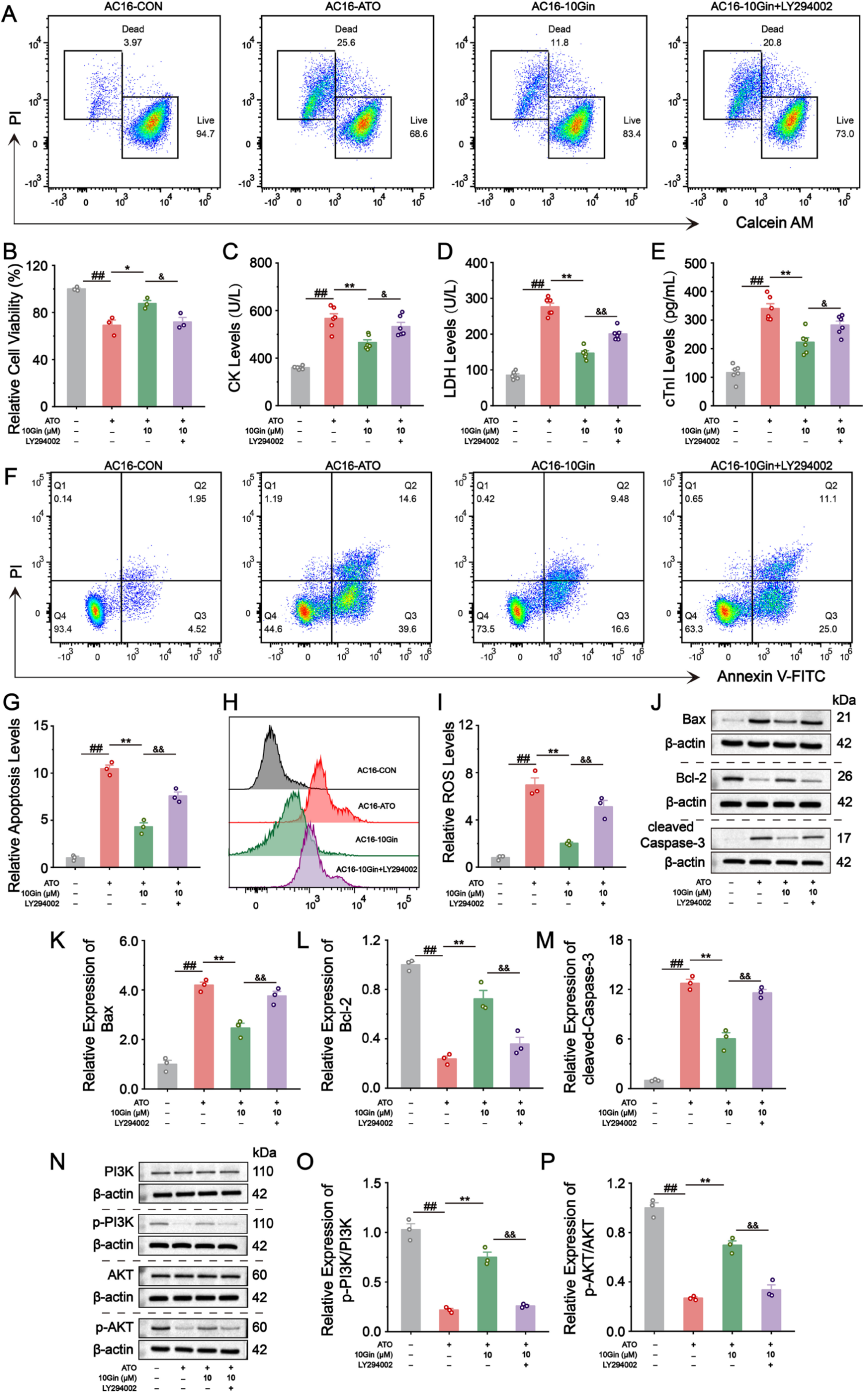
10Gin protects AC16 cells against ATO‐induced injury through activation of the PI3K/AKT signaling pathway. (A, B) Representative flow cytometry images of Calcein‐AM/PI double staining and quantitative analysis of relative cell viability (*n* = 3). (C–E) Cell injury biomarkers CK, LDH, and cTnI levels in the cell supernatant (*n* = 6). (F, G) Representative flow cytometry images of apoptosis and quantitative analysis of relative apoptosis levels (*n* = 3). (H, I) Representative flow cytometry images of ROS and quantitative analysis of relative ROS levels (*n* = 3). (J) Representative WB bands of Bax, Bcl‐2, and cleaved Caspase‐3. (K–M) Quantitative analyses of Bax, Bcl‐2, and cleaved Caspase‐3 protein expression, respectively (*n* = 3). (N) Representative WB bands of PI3K, p‐PI3K, AKT, and p‐AKT. (O, P) Quantitative analyses of the p‐PI3K/PI3K and p‐AKT/AKT ratios (*n* = 3). Data are represented as mean ± SEM. ^##^ *p* < 0.01 vs. AC16‐CON; *p < 0.05, **p < 0.01 vs. AC16‐ATO; ^&^ *p* < 0.05, ^&&^ *p* < 0.01 vs. AC16‐10Gin.

To further evaluate the cardioprotection of 10Gin in human‐derived cardiomyocytes, this study additionally used AC16 cells for verification. As shown in Figure 8, compared with the AC16‐CON group, ATO treatment significantly decreased AC16 cell viability and markedly elevated the levels of apoptosis, ROS, CK, LDH, and cTnI (*p* < 0.01), indicating that ATO toxicity can impair AC16 cells. In contrast, 10Gin treatment significantly enhanced cell viability and attenuated ATO‐induced apoptosis, oxidative stress, and the levels of myocardial injury markers (*p* < 0.05 or *p* < 0.01). Notably, LY294002 pretreatment partially attenuated the protective effects of 10Gin (*p* < 0.05 or *p* < 0.01). WB results further showed that ATO significantly decreased the p‐PI3K/PI3K and p‐AKT/AKT ratios in AC16 cells, upregulated the expression levels of Bax and cleaved Caspase‐3, and downregulated Bcl‐2 expression (*p* < 0.01). 10Gin treatment reversed these ATO‐induced alterations, whereas pretreatment with the PI3K inhibitor LY294002 attenuated the regulatory effects of 10Gin on PI3K/AKT phosphorylation and apoptosis‐related proteins (*p* < 0.01). These results in AC16 cells were consistent with those obtained in H9c2 cells, further validating the cardioprotective role of 10Gin against ATO‐induced HI.

## 4 Discussion

ATO remains a cornerstone of first‐line treatment for APL and has also shown anti‐tumor activity in several solid tumors (Chen et al. 2023; Pollyea et al. 2023). However, its dose‐dependent and life‐threatening cardiotoxicity remains a major barrier to its wider clinical application (Yan et al. 2024). Although oxidative stress, inflammatory cascades, cardiomyocyte apoptosis, and ion channel dysfunction have been implicated in ATO‐induced HI, there are currently no clinically approved cardioprotective agents specifically indicated for patients receiving ATO therapy (Cui et al. 2024; Wang et al. 2023). 10Gin is a key bioactive constituent from ginger. In recent years, it has garnered growing attention owing to its potent antioxidant, anti‐inflammatory, and anti‐apoptotic properties, which are partially ascribed to its longer alkyl side chain structure relative to other gingerol analogs (Garza‐Cadena et al. 2023). In the present study, we integrated NP, an in vivo mouse model, and in vitro cell experiments to systematically demonstrate that 10Gin alleviates ATO‐induced HI and to elucidate its underlying mechanisms.

In terms of animal experimental phenotypes, the in vivo data demonstrated that 10Gin ameliorated ATO‐induced HI, including myocardial structural damage, fibrosis, elevated serum myocardial injury markers, and cardiac contractile dysfunction (Figures 2 and 3). These findings are consistent with previous studies demonstrating that ATO can induce myocardial histopathological damage and fibrosis (Liang et al. 2020). The reductions in CK, LDH, and the highly sensitive and specific myocardial injury markers cTnT and cTnI after 10Gin intervention further confirmed its cardioprotective effect (Sörensen et al. 2023). Moreover, ECHO findings confirmed that ATO induced severe impairment of LV systolic function in mice, as evidenced by significant reductions in EF and FS. This aligns closely with the clinical observations that ATO chemotherapy is associated with decreased cardiac function and even progression to heart failure in patients (Wang et al. 2023; Yan et al. 2024). In contrast, 10Gin intervention significantly improved the cardiac function parameters and effectively improved LV systolic function (Figure 3). Therefore, the cardioprotective effect of 10Gin was supported by histological, biochemical, and cardiac functional evidence.

NP analysis provided mechanistic clues for the cardioprotective effect of 10Gin against ATO‐induced HI. In the present study, we identified a total of 32 potential therapeutic targets of 10Gin against ATO‐induced HI, among which AKT1, CASP3, IL6, PPARG, and ESR1 were defined as the five highest‐ranked hub targets. GO and KEGG enrichment analyses revealed that these targets are primarily involved in apoptosis, inflammatory response, oxidative stress, transcriptional regulation, kinase activity, and the PI3K/AKT signaling pathway (Figure 1). Previous studies have shown that ATO promotes cardiomyocyte apoptosis through CASP3 activation, induces inflammatory injury through IL‐6 upregulation, inhibits the AKT1‐mediated pro‐survival signaling, and exacerbates oxidative stress through PPARG‐associated mechanisms (Li, Ma, et al. 2022; Qin et al. 2024; Rao et al. 2023; Thangapandiyan et al. 2024). Furthermore, inhibition of ESR1 is also recognized to be associated with exacerbated cardiac injury (Chen et al. 2025). AKT1 is a key serine/threonine kinase in the PI3K/AKT pathway, which plays a critical role in maintaining cardiac homeostasis and mediating stress responses (Ghafouri‐Fard et al. 2022; Kumar et al. 2025). By integrating these core targets with the pathway enrichment results, the PI3K/AKT signaling pathway was selected as a major candidate pathway for experimental validation. Consistent with the NP predictions, our experimental data demonstrated that ATO suppresses the phosphorylation levels of PI3K and AKT in myocardial tissues, H9c2 cells, and AC16 cells, whereas 10Gin restored the p‐PI3K/PI3K and p‐AKT/AKT ratios (Figures 5, 7, and 8). Notably, the PI3K inhibitor LY294002 partially attenuated the protective effects of 10Gin on cell viability, myocardial injury markers, oxidative stress, inflammatory cytokine release, and apoptosis, further supporting the involvement of PI3K/AKT pathway activation in 10Gin‐mediated cardioprotection (Figures 6, 7, 8).

The PI3K/AKT signaling pathway is a well‐established key regulator of cardiomyocyte survival, homeostasis, and stress responses, and its dysregulation has been associated with cardiovascular injury, including cardiotoxicity caused by chemotherapy (Deng and Liu 2022). In the present study, 10Gin attenuated ATO‐induced accumulation of ROS and MDA while enhancing the activities of antioxidant enzymes including SOD, CAT, and GSH‐Px in mouse myocardial tissue (Figure 3). In H9c2 cells, 10Gin reduced ROS levels and MDA content and restored SOD and GSH‐Px activities, whereas in AC16 cells, it significantly reduced ROS production (Figures 6 and 8). These effects may be associated with downstream antioxidant signaling pathways modulated by the PI3K/AKT pathway. As shown in previous studies, activated AKT can promote nuclear factor erythroid 2‐related factor 2 (Nrf2) nuclear translocation and enhance the transcription of antioxidant and detoxification enzymes (Lv et al. 2020; Yu and Xiao 2021). ATO exposure has also been reported to suppress PI3K/AKT phosphorylation and downstream Nrf2 activation in cardiomyocytes (Thangapandiyan et al. 2024). Although the present study did not directly assess Nrf2 activation, our results suggest that restoration of the PI3K/AKT pathway may contribute to the antioxidant effects of 10Gin against ATO‐induced HI.

Inflammation is another major contributor to ATO‐induced HI. Oxidative stress and inflammatory responses can form a self‐reinforcing cycle, which in turn exacerbates myocardial injury (Gambini and Stromsnes 2022; Zhang et al. 2018). Excessive ROS production caused by ATO may activate the NLRP3 inflammasome and NF‐κB pathway, thereby facilitating the release of pro‐inflammatory cytokines including IL‐6, TNF‐α, and IL‐1β (Ahmed et al. 2025; Gan et al. 2023). Meanwhile, these inflammatory mediators can further enhance ROS generation and aggravate myocardial injury. Activated AKT has been reported to suppress NF‐κB nuclear translocation and attenuate the transcriptional activation of pro‐inflammatory cytokines (Higuchi et al. 2006). In the present study, 10Gin decreased IL‐1β, IL‐6, and TNF‐α concentrations and mRNA expression levels in myocardial tissue and reduced the concentrations of these cytokines in H9c2 cell culture supernatants (Figures 4 and 7). Notably, LY294002 pretreatment weakened the anti‐inflammatory effect of 10Gin in H9c2 cells. These findings indicate that PI3K/AKT activation is involved in the anti‐inflammatory action of 10Gin.

The anti‐apoptotic effects of 10Gin also appear to be closely associated with the activation of the PI3K/AKT pathway. ATO‐induced cardiomyocyte apoptosis has been associated with the activation of the mitochondrial intrinsic apoptotic pathway, which involves alterations in Bcl‐2 family protein expression, increased mitochondrial membrane permeability, cytochrome c release, and subsequent caspase activation (Czabotar and Garcia‐Saez 2023; Green 2022). Activated AKT can attenuate mitochondrial apoptosis by modulating apoptosis‐related proteins, including restoration of the Bcl‐2/Bax homeostasis and inhibition of Caspase‐3 activation, thereby preserving mitochondrial membrane integrity and reducing cardiomyocyte apoptosis (Bromage et al. 2019; Simonyan et al. 2016; Zhou et al. 2024). In the present study, 10Gin increased Bcl‐2 expression, decreased Bax and cleaved Caspase‐3 protein levels, reduced the percentage of TUNEL‐positive nuclei in myocardial tissue, and attenuated apoptosis in H9c2 and AC16 cells (Figures 4, 7, and 8). LY294002 pretreatment partially weakened the anti‐apoptotic effects of 10Gin in both cell models. These results further support that 10Gin exerts anti‐apoptotic effects by activating the PI3K/AKT pathway.

From a clinical perspective, 10Gin has potential clinical utility for patients with APL or other malignancies receiving ATO‐based chemotherapy. However, the present results should not be interpreted as direct evidence that 10Gin can be used as a dietary supplement during ATO therapy. Several limitations must be acknowledged to enable an objective interpretation of the results. Although LY294002 has been employed to pharmacologically validate the involvement of the PI3K/AKT signaling pathway in the cardioprotection of 10Gin, the causal role of this pathway remains unconfirmed through genetic approaches. Additionally, the downstream regulatory effects of PI3K/AKT on Nrf2 nuclear translocation and NF‐κB activity have not been directly verified experimentally, and the corresponding mechanistic inferences are based on published evidence. The effective dosage, pharmacokinetic profiles, tissue distribution, and safety profile of 10Gin in humans remain to be fully elucidated. Notably, given that the anti‐leukemic activity of ATO is partially mediated by oxidative stress and apoptosis, it remains critical to further determine whether 10Gin may interfere with the therapeutic efficacy of ATO. Although 10Gin has shown anti‐tumor potential (Liang et al. 2023), its interaction with ATO in leukemia cells remains to be fully characterized. Therefore, prior to consideration of its clinical translation, further investigations are warranted in APL cells and leukemia xenograft animal models, coupled with the optimization of relevant pharmacokinetic and safety assessments.

## 5 Conclusions

**FIGURE 9:**
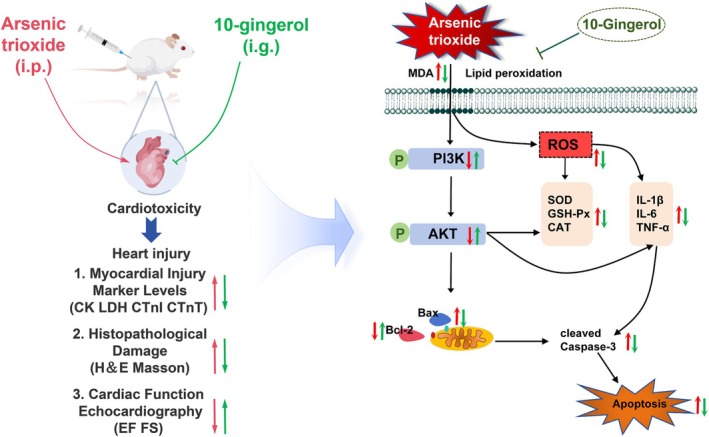
Mechanism diagram illustrating the protection of 10Gin against ATO‐induced HI.

This study integrated NP analysis with in vitro and in vivo experimental verification and demonstrated that 10Gin exerted antioxidant, anti‐inflammatory, and anti‐apoptotic effects by activating the PI3K/AKT signaling pathway, thereby alleviating ATO‐induced myocardial structural damage, cardiac dysfunction, and myocardial cytotoxicity (Figure 9). Collectively, this study presents 10Gin as a potential adjuvant candidate for mitigating ATO‐induced adverse cardiac effects in patients with APL or other malignancies undergoing ATO‐based chemotherapy. However, it should be noted that the current findings are derived from fundamental research, and the clinical efficacy of 10Gin warrants further investigation.

## Author Contributions

**Hui Yin:** investigation, data curation, formal analysis, validation. **Bin Zheng:** data curation, formal analysis, writing – original draft, validation. **Jiabao Chen:** methodology, formal analysis. **Chunhua Li:** writing – review and editing, project administration. **Lan Yan:** conceptualization. **Hefei Wang:** writing – review and editing, software. **Hua Zhou:** project administration, conceptualization. **Xiaodong Xin:** visualization, resources.

## Funding

The experiment was supported by the Research Foundation of Administration of Traditional Chinese Medicine of Hebei Province, China (No. 2025015), the Innovation Funding Project of Hebei University of Traditional Chinese Medicine (XCXZZBS2026027), and the Li Chunhua National Famous TCM Experts Inheritance Studio.

## Ethics Statement

The Laboratory Animal Welfare Ethics Committee of Hebei University of Chinese Medicine oversaw the ethical conduct of all animal experiments. The study protocols were approved by the Laboratory Animal Welfare Ethics Committee of Hebei University of Chinese Medicine (Approval No: DWLL202506003).

## Conflicts of Interest

The authors declare no conflicts of interest.

## Supporting information


**Table S1:** Summary of uniform resource locators used in this study.
**Table S2:** List of antibodies used.
**Table S3:** CCK‐8 assay data used for determining the optimal concentrations of ATO and 10Gin in H9c2 cells.

## Data Availability

The data that support the findings of this study are available from the corresponding author upon reasonable request.
