# Correction to “10‐Gingerol Alleviates Arsenic Trioxide‐Induced Cardiotoxicity: Mechanisms Involving the PI3K/AKT Pathway Revealed by Network Pharmacology and Experimental Validation”

**DOI:** 10.1002/fsn3.72505

**Published:** 2026-10-20

Zheng, B. , H. Yin , J. Chen , X. Xin , L. Yan , C. Li , H. Wang , and H. Zhou . 2026. “10‐Gingerol Alleviates Arsenic Trioxide‐Induced Cardiotoxicity: Mechanisms Involving the PI3K/AKT Pathway Revealed by Network Pharmacology and Experimental Validation.” Food Science & Nutrition 14, no. 8: e72180.10.1002/fsn3.72180PMC1341598542528963

In Figure 6N, the MERGE image for the C‐CON group was incorrectly inserted during figure assembly and did not correspond to the DAPI and DCFH‐DA single‐channel images shown in the same column. Accordingly, this image has been replaced with the correct MERGE image generated from the corresponding DAPI and DCFH‐DA single‐channel images. The corrected Figure 6 is provided below.

**FIGURE 6:**
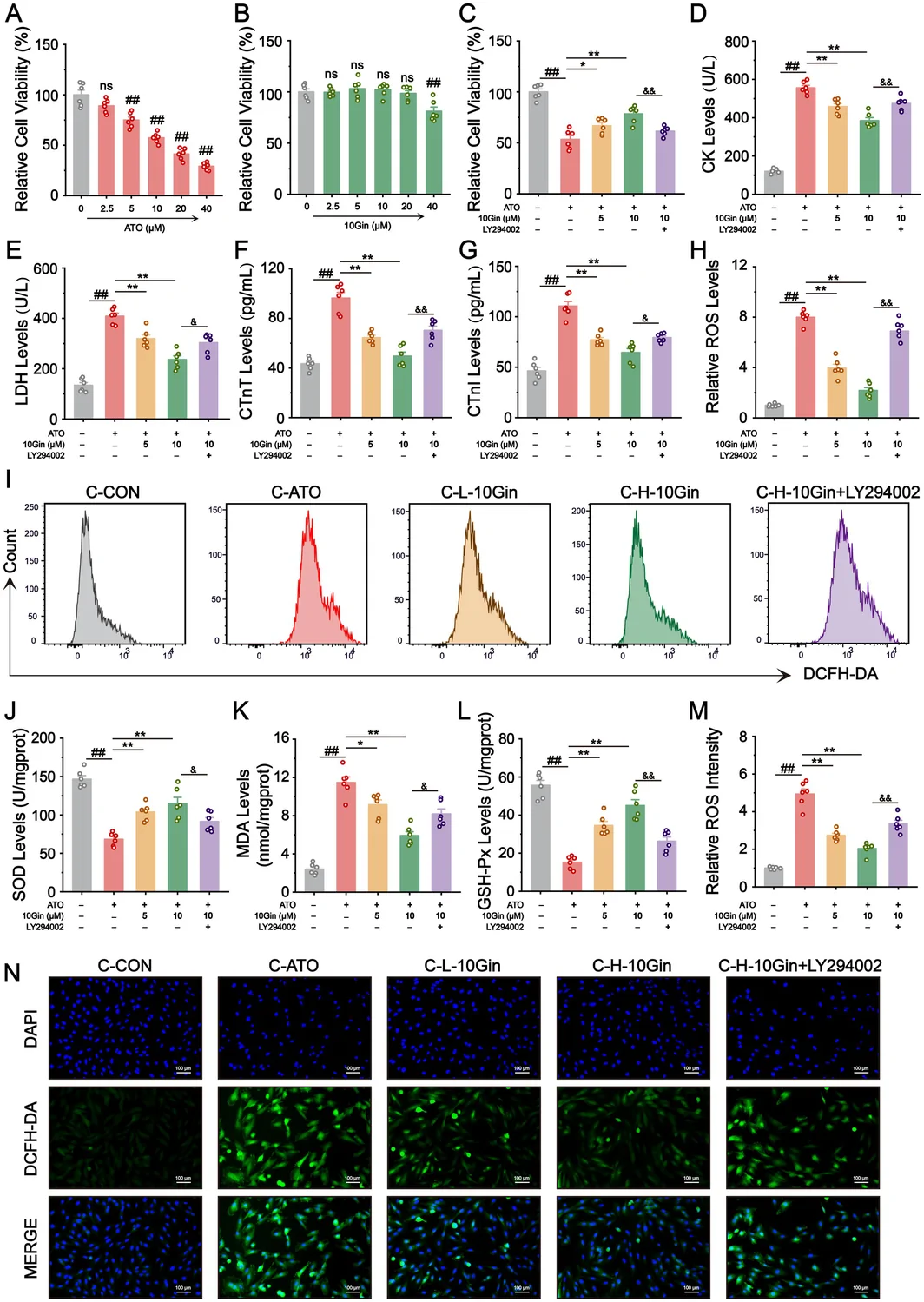
10Gin attenuates ATO‐induced H9c2 cell injury by enhancing cell viability, reducing myocardial biomarkers, and mitigating oxidative stress levels. (A–B) Different concentrations of ATO (2.5–40 μM) and 10Gin (2.5–40 μM) on the viability of H9c2 cells (*n* = 6). (C) Effects of 10Gin and LY294002 on the viability of ATO‐injured H9c2 cells (*n* = 6). (D–G) Cell injury biomarkers CK, LDH, cTnT, and cTnI levels in the cell supernatant (*n* = 6). (H–I) Representative flow cytometry images of ROS and relative ROS levels (n = 6). (J–L) Intracellular SOD and GSH‐Px activities, and MDA content (*n* = 6). (M–N) Representative DCFH‐DA staining images and relative ROS intensity (200×, scale bar = 100 μm) (*n* = 6). Data are represented as mean ± SEM. ns, no significance, ^##^ *p* < 0.01 vs. C‐CON; *p < 0.05, **p < 0.01 vs. C‐ATO; ^&^ *p* < 0.05, ^&&^ *p* < 0.01 vs. C‐H‐10Gin.

We apologize for this error.

