# Supplementary material for: 10‐Gingerol Alleviates Arsenic Trioxide‐Induced Cardiotoxicity: Mechanisms Involving the PI3K/AKT Pathway Revealed by Network Pharmacology and Experimental Validation
Source: Food Sci Nutr. 2026 Jul 28;14(8):e72180. doi: 10.1002/fsn3.72180 (PMC13415985; doi:10.1002/fsn3.72180)
Supplement: Supplementary file 1 — Table S1: Summary of uniform resource locators used in this study. Table S2: List of antibodies used. Table S3: CCK‐8 assay data used for determining the optimal concentrations of ATO and 10Gin in H9c2 cells. [file FSN3-14-e72180-s001.docx]

**Supplementary Material 1**

**Table S1.** Summary of uniform resource locators used in this study.

| **Official Name of The Database** | **Uniform Resource Locator** |
| --- | --- |
| TCMSP | http://www.tcmsp-e.com/ |
| STP | http://www.swisstargetprediction.ch/ |
| SPTP | https://prediction.charite.de/subpages/target_prediction.php |
| PubChem | https://pubchem.ncbi.nlm.nih.gov/ |
| CTD | https://ctdbase.org/ |
| GeneCards | https://www.genecards.org/ |
| OMIM | https://www.omim.org/ |
| DrugBank | https://go.drugbank.com/ |
| UniProt | https://www.uniprot.org/ |
| String | https://cn.string-db.org/ |
| DAVID | https://davidbioinformatics.nih.gov/ |

TCMSP: Traditional Chinese Medicine Systems Pharmacology; STP: Swiss Target Prediction; SPTP: Super-PRED Target Prediction; CTD: Comparative Toxicogenomics Database; DAVID: Database for Annotation, Visualization, and Integrated Discovery.

**Table S2.** List of antibodies used.

| **Antibodies** | **Manufacturer** | **Catalog number** | **Dilution ratio** | **Kda** |
| --- | --- | --- | --- | --- |
| Anti-Bcl-2 | Affinity | BF9103 | 1:1000 | 26 |
| Anti-Bax | Servicebio | GB11690 | 1:1000 | 21 |
| Anti-cleaved Caspase-3 | Abcam | AB2302 | 1:1000 | 17 |
| Anti-PI3K | Abcam | ab151549 | 1:1000 | 110 |
| Anti-p-PI3K | BIOSS | BS-5570R | 1:1000 | 110 |
| Anti-AKT | Servicebio | GB11689 | 1:1000 | 60 |
| Anti-p-AKT | Affinity | AF0908 | 1:1000 | 60 |
| Anti-β-actin | Servicebio | GB12001 | 1:3000 | 42 |

**Table S3.** CCK-8 assay data used for determining the optimal concentrations of ATO and 10Gin in H9c2 cells.

| **Group** | **Treatment Concentration** | **Cell viability (%, MEAN ± SEM )** |
| --- | --- | --- |
| ATO | 0 μM | 100.00 ± 4.67 |
| ATO | 2.5 μM | 88.90 ± 2.79 |
| ATO | 5 μM | 75.17 ± 3.08 |
| ATO | 10 μM | 57.54 ± 1.95 |
| ATO | 20 μM | 41.32 ± 2.44 |
| ATO | 40 μM | 29.10 ± 1.46 |
| 10Gin | 0 μM | 100.00 ± 2.95 |
| 10Gin | 2.5 μM | 99.86 ± 1.63 |
| 10Gin | 5 μM | 103.12 ± 3.73 |
| 10Gin | 10 μM | 102.18 ± 2.58 |
| 10Gin | 20 μM | 98.52 ± 2.76 |
| 10Gin | 40 μM | 81.15 ± 4.09 |

**2.4.6. Flow Cytometric**

Apoptosis and intracellular ROS levels in H9c2 cells were assessed via flow cytometry. For apoptosis analysis, cells were stained with an Annexin V-FITC/PI apoptosis detection kit (AP101, Lianke Biology, China), while ROS detection utilized the DCFH-DA probe. H9c2 cells were inoculated in 6-well plates. After grouping and treatment, the adherent and suspended cells were collected, and washed twice with PBS by centrifugation. After resuspending the cells in 1× Binding Buffer for apoptosis detection, blank and single-stained control tubes were prepared to calibrate the flow cytometer. Annexin V-FITC and PI were added to the cell suspensions and incubated at room temperature in the dark for 5 min. Subsequently, the samples were analyzed by flow cytometry, and apoptosis rates were quantified using FlowJo software. The ROS detection was performed by diluting DCFH-DA in medium to 10 μM, incubating at 37 °C in the dark for 20 min. During this period, the cell suspensions were gently mixed. After washing, the relative fluorescence intensity was detected at 488 nm excitation and 525 nm emission. This assay included 6 biological replicates (n = 6).
